# An Interpretable Machine Learning Model for Predicting the Presence of Talaromycosis in HIV Patients Lacking Skin Lesions

**DOI:** 10.1007/s11046-026-01089-y

**Published:** 2026-07-21

**Authors:** Jiaguang Hu, Wenming He, Qun Tian, Yanqiu Lu, Peng Zhang, Jinyu Qin, Chuan Qin, Ying Wu, Cheng Huang, Xu Li, Luhuai Feng, Linghua Li, Zhongsheng Jiang, Jianning Jiang

**Affiliations:** 1 Department of Infectious Diseases, The First Affiliated Hospital of Guangxi Medical University, Nanning, 530021 Guangxi China; 2 Division of Infectious Diseases, Liuzhou People’s Hospital Affiliated to Guangxi Medical University, Liuzhou, Guangxi China; 3 Department of Infection Control Management, Liuzhou People’s Hospital Affiliated to Guangxi Medical University, Liuzhou, Guangxi China; 4 Liuzhou Key Laboratory of Infection Disease and Immunology, Liuzhou People’s Hospital Affiliated to Guangxi Medical University, Liuzhou, Guangxi China; 5 Clinical Research Center, Chongqing Public Health Medical Center, Shapingba, China; 6 Infectious Disease Center, Guangzhou Eighth People’s Hospital, Guangzhou Medical University, Guangzhou, China; 7 Internal Medicine Ward One, The Third People’s Hospital of Guilin, Guilin, Guangxi China; 8 Oncology Department, Liuzhou People’s Hospital Affiliated to Guangxi Medical University, Liuzhou, Guangxi China; 9 Liuzhou Key Laboratory of Severe Abdominal Infection Research, Liuzhou, Guangxi China; 10 Guangxi Key Laboratory of Clinical Disease Biotechnology Research, Liuzhou People’s Hospital, Liuzhou, Guangxi China; 11 Department of Endocrinology and Metabolism, Nephrology, Guangxi Medical University Cancer Hospital, Nanning, China; 12 Key Laboratory of Early Prevention and Treatment for Regional High-Frequency Tumor (Guangxi Medical University), Ministry of Education, Nanning, Guangxi 530021 People’s Republic of China

**Keywords:** HIV infection, Talaromycosis, Machine learning, Diagnostic model, Data visualization

## Abstract

**Introduction:**

The existing predictive models for talaromycosis in people living with HIV without skin lesions are limited by established risk factors and traditional statistical approaches. This study aims to develop an interpretable machine learning(ML) model for predicting the presence of talaromycosis in HIV patients without skin lesions and to validate its clinical applicability.

**Methods:**

This retrospective multicenter study involved the analysis of electronic medical records from four tertiary hospitals in China, covering the period from 2010 to 2019. The training dataset comprised 1009 HIV patients with opportunistic infections, while external validation was conducted using data from 305 patients at an independent center. From an initial set of 36 variables, twelve key features were selected, including albumin, absolute lymphocyte count, hemoglobin, alanine aminotransferase (ALT), aspartate aminotransferase (AST), AST/ALT ratio, C-reactive protein, white blood cell count, platelet count, peripheral or abdominal lymphadenopathy, CD4^+^ T-cell count, and age. Five ML algorithms were evaluated using tenfold cross-validation. Model performance was measured using the AUC, ACC, and F1-score. Calibration curves and decision curve analysis (DCA) were employed to assess the model’s reliability and clinical net benefit. The optimal model was subsequently implemented as a web-based tool.

**Results:**

The Support Vector Machine (SVM) exhibited superior performance compared to other models, achieving an AUC of 0.809 (95% CI 0.778–0.838), an ACC of 0.714, and an F1-score of 0.689. External validation demonstrated enhanced performance metrics, with an AUC of 0.921 (95% CI 0.889–0.951), ACC of 0.853, and an F1-score of 0.819. DCA indicated a significant net clinical benefit across various risk thresholds, and calibration curves showed strong concordance between predicted and observed risks.

**Conclusion:**

The interpretable SVM model effectively stratifies the risk of talaromycosis in people living with HIV without skin lesions in endemic regions, aligning with WHO recommendations for early diagnosis and treatment of priority fungal pathogens. Its integration into a web-based tool enhances clinical accessibility for early intervention in resource-constrained settings.

**Supplementary Information:**

The online version contains supplementary material available at 10.1007/s11046-026-01089-y.

## Introduction

Talaromycosis is classified as an invasive fungal infection, predominantly affecting individuals with weakened immune systems, particularly people living with human immunodeficiency virus (HIV). It is widespread in Asia’s tropical and subtropical regions, notably in Southeast Asian nations like Vietnam, Thailand, and southern China [1]. In Southeast Asia, it accounts for between 6.4% and 11% of hospital admissions related to HIV in Vietnam and 3.3% in Thailand. In southern China, the prevalence is higher, with Guangxi and Guangdong reporting rates of 16.1% and 17.3%, respectively, highlighting its significant burden in these areas [1–6]. Despite the implementation of antifungal therapy, delayed diagnosis contributes to a high in-hospital mortality rate among patients with talaromycosis, ranging from 16.7% to 30% [7–9]. However, the efficacy of these interventions relies on the timely diagnosis of infected individuals.

The typical clinical features of *Talaromyces marneffei (*TM) infection commonly include fever, weight loss, lower Hemoglobin (HB) levels, generalized fatigue, skin lesions, peripheral or abdominal lymphadenopathy (POAL), and hepatosplenomegaly [10]. Studies have demonstrated that skin lesions of TM infection often present as central umbilicated lesions, which can serve as critical diagnostic indicators for early detection. Approximately one-third of patients with TM infection exhibit skin lesions [11]. Microbiological culture is currently considered the definitive standard for diagnosing talaromycosis. However, this method is time-consuming, and the isolation or identification of pathogens from clinical specimens often takes more than 10 days. Conversely, a presumptive diagnosis of *Talaromyces marneffei* can be swiftly established by identifying its distinctive morphological features in biopsy tissue histopathology or peripheral blood smears in patients with disseminated infection. This method facilitates the earlier commencement of antifungal therapy, which is crucial for enhancing patient outcomes[12–14]. Despite these advantages, invasive tests and false-negative results remain a challenge for the prompt identification of TM infection, particularly in patients lacking skin lesions or with low fungal burden [13].

Early initiation of antifungal therapy in talaromycosis can significantly reduce mortality and mitigate the severity of the disease [7, 15–17]. However, the efficacy of targeted interventions depends on the timely identification of patients with active infection. Although recent studies have used dynamic nomograms to evaluate the potential for TM infection in hospitalized people living with HIV, relying on skin lesions as a risk factor may reduce diagnostic accuracy (ACC) for those without skin lesions [18]. There is a critical demand for quantitative, accessible, and efficient approaches to evaluate the presence of talaromycosis in people living with HIV who lack skin lesions.

In recent years, machine learning (ML) techniques utilizing electronic medical records (EMR) have gained significant traction and acceptance among healthcare professionals. Unlike conventional statistical approaches, ML algorithms impose fewer limitations on data requirements and excel at modeling intricate datasets [19], driving their growing application in the medical field. However, the inherent complexity of ML models, especially their “opaque system” nature, often limits transparency in understanding their decision-making processes [20]. To overcome this limitation, model interpretation tools are essential for elucidating the underlying mechanisms of ML models. This study utilized the Shapley Additive Explanations (SHAP) methodology to develop an interpretable model. This tool enhances the interpretability and clinical utility of ML-based predictions by clearly visualizing each variable’s contribution to the model’s outcomes. Additionally, this study developed an accessible web-based application for implementing the predictive model, enabling clinicians to utilize the model for diagnostic prediction without necessitating the installation of Python or the acquisition of programming skills.

## Methods

### Design of the Study

This study analyzed data from a multicenter retrospective cohort of people living with HIV in China, including those hospitalized at Liuzhou People’s Hospital, the Third People’s Hospital of Guilin, Guangzhou Eighth People’s Hospital, and Chongqing Public Health Medical Center. The model development dataset comprised 751 patients from Liuzhou People’s Hospital, 122 from Chongqing Public Health Medical Center, and 136 from Guangzhou Eighth People’s Hospital, spanning August 3, 2010, to January 17, 2019. The external validation cohort comprising 305 cases was retrospectively collected from the Third People’s Hospital of Guilin between December 16, 2015, and December 12, 2018. This study is divided into three main phases:(1) selecting participants and screening variables; (2) developing and evaluating models; and (3) creating a web application for the best model. The Ethics Committee of the Chongqing Public Health Medical Center (2019-003-02-KY) approved the study. The ethics committees waived the need for informed consent from participants due to the study’s retrospective and anonymized nature. Eligible participants included individuals aged 18 years or older who had a confirmed diagnosis of HIV. Additionally, participants were required to have a CD4^+^ T-cell count of less than 200 cells/μL at the time of admission and to have been hospitalized for more than three days due to suspected opportunistic infections. Exclusion criteria included pregnancy, lactation, and the presence of skin lesions, and the study excluded medical records that were incomplete (> 20% missing data). The study protocol adhered to the TRIPOD + AI reporting guidelines for clinical prediction models [21], and a completed checklist is available in Supplementary Material 1.

### Outcomes

The diagnostic model’s outcome variable determined the presence of talaromycosis in patients. Talaromycosis was diagnosed when TM was isolated from clinical specimens using standard culture techniques [12] or identified in biopsy tissue histopathology.

### Sample size Calculation

The sample size was determined using the events per variable (EPV) metric [22, 23], a widely recognized approach in statistical analyses. In southern China, the incidence of HIV co-infection with *Talaromyces marneffei* is 0.16. Considering our objective to include twelve predictor variables and establish the EPV at 10, the required sample size was calculated using the following formula:

$$ {\mathrm{Sample}}\;{\mathrm{size}}{\kern 1pt} = \frac{{{\mathrm{Number}}\;{\mathrm{of}}\;{\mathrm{variables}} \times {\mathrm{EPV}}}}{{1 - {\mathrm{Incidence}}\;{\mathrm{rate}}}} $$

$$ = \frac{12 \times 10}{{1 - 0.16}} = 143 $$

### Data Collection and Model Predictors

The patient data were retrospectively extracted from hospital admission records covering the period from August 3, 2010, to January 17, 2019. Missing data in the clinical records were managed through mean substitution. Initially, our model was developed through a systematic review, meta-analysis, and expert consensus, incorporating a comprehensive array of variables. These variables encompassed the presence of oral candidiasis, tuberculosis, bacterial pneumonia, pneumocystis pneumonia, cryptococcosis, cytomegalovirus disease, herpes simplex virus disease, and lymphoma. Additionally, demographic and clinical factors such as sex, age, body mass index (BMI), nationality, occupation, marital status, and injection drug use were included. The model also considered clinical symptoms and laboratory findings, including fever, cough, poor appetite, hepatomegaly, splenomegaly, HB levels, white blood cell count (WBC), platelet count (PLT), absolute lymphocyte count (ALC), C-reactive protein (CRP), AST, ALT, AST/ALT ratio, albumin (ALB), blood urea nitrogen (BUN), creatinine (Cr), (1–3)-β-D glucan (G) levels, CD4^+^ T-cell count(CD4), and the presence of hepatitis C and hepatitis B. All predictor variables were assessed utilizing standard laboratory techniques upon hospital admission, typically within the initial 24 h. Specific definitions were employed: fever was characterized by an axillary temperature of ≥ 37.5 °C, while hepatomegaly, splenomegaly, and lymphadenopathy were identified through physical examination or imaging modalities. Laboratory parameters were quantified using automated analyzers, such as the Sysmex XN-9000 for hematological assessments and the Roche Cobas c702 for biochemical analyses. Given the retrospective nature of the study, assessors were not blinded to patient outcomes; however, all measurements were conducted as part of routine clinical care before the confirmation of outcomes. The attending physicians documented physical examination findings, such as peripheral or abdominal lymphadenopathy, hepatomegaly, and splenomegaly, upon patient admission. Each physician possessed a minimum of three years of clinical experience in the field of infectious diseases. Due to the retrospective nature of the study, a formal assessment of inter-rater reliability was not conducted, and demographic information regarding the assessors was not collected. Symptoms, including fever, cough, and poor appetite, were recorded by nursing staff or physicians based on patient self-reports and clinical records. Laboratory parameters were generated automatically by standard hospital analyzers, eliminating the need for subjective interpretation.

To ensure robustness in our analysis, we utilized five complementary feature selection techniques: Least Absolute Shrinkage and Selection Operator (Lasso) regression, Random Forest (RF), Boruta, Extreme Gradient Boosting (XGBoost), and Mutual Information (MI). These methods were chosen to effectively capture both linear and non-linear relationships, mitigate overfitting, and improve generalizability. Comprehensive descriptions of each algorithm, along with parameter settings and selection outcomes, are available in the Supplementary Methods 1.

### Statistical Analysis

Demographic, clinical, and laboratory data from the derivation cohort were analyzed upon hospital admission. Continuous variables are reported as medians with interquartile ranges (IQRs) and categorical variables as frequencies and percentages. A two-sided *p*-value of less than 0.05 was used to establish statistical significance. The gaze function in the pandas package enhances data analysis efficiency in medical research by automatically identifying variable types, such as continuous or categorical, and applying appropriate statistical methods to generate comprehensive descriptive statistics. Data analyses were performed utilizing SPSS version 29.0 (IBM Analytics, USA) and Anaconda 3, incorporating Python version 3.12.

### Model Development and Comparison

In the study’s final cohort of 1314 patients, 379 had missing data in variables such as BMI (198), CRP (7), ALB (1), BUN (8), Cr (8), and G-test (157). These missing values were random. We used the pandas library to handle this by imputing the mean for each column and applying the fill () method for data preprocessing. The dataset of 1009 patients was divided into 80% training and 20% testing subsets using the Pandas library for internal validation. Preprocessing steps included encoding categorical variables as binary indicator variables, eliminating features with minimal variance, and standardizing continuous variables to minimize overfitting. Subsequently, a prediction model was built based on the identified predictor variables. Eight machine learning models—logistic regression (LR), SVM, RF, multi-layer perceptron (MLP), naive Bayes, decision tree (DT), K-nearest neighbor (KNN), and XGBoost—were utilized to assess talaromycosis risk. The models were assessed and compared through the use of accuracy (ACC), area under the receiver operating characteristic curve (AUC), specificity, sensitivity, positive predictive value (PPV), negative predictive value (NPV), F1 score, and Matthews Correlation Coefficient (MCC) metrics. These metrics were similarly employed to assess the efficacy of the optimal model’s performance in external validation. Calibration curves were utilized to assess the predictive performance of the optimal model, whereas clinical decision curve analysis (DCA) was employed to evaluate its clinical utility.

### Interpretation of the Model and Its Application Within the Network

Interpretability refers to the clarification of how ML models generate outcomes. The inherent opacity of machine learning models often hinders their clinical application, prompting significant research to improve interpretability [24, 25]. This study presents an intuitive model explanation framework employing the SHAP (Shapley Additive exPlanations) BreakDown package, which quantifies the extent of individual predictors’ contributions to model predictions. Furthermore, we developed an intuitive model interpreter that works independently of any specific model and uses input predictor variables to interpret results. The Gradio package facilitated the incorporation of predictive variables and optimal modeling into an interactive web application.

## Results

### Demographic Attributes

**Fig. 1:**
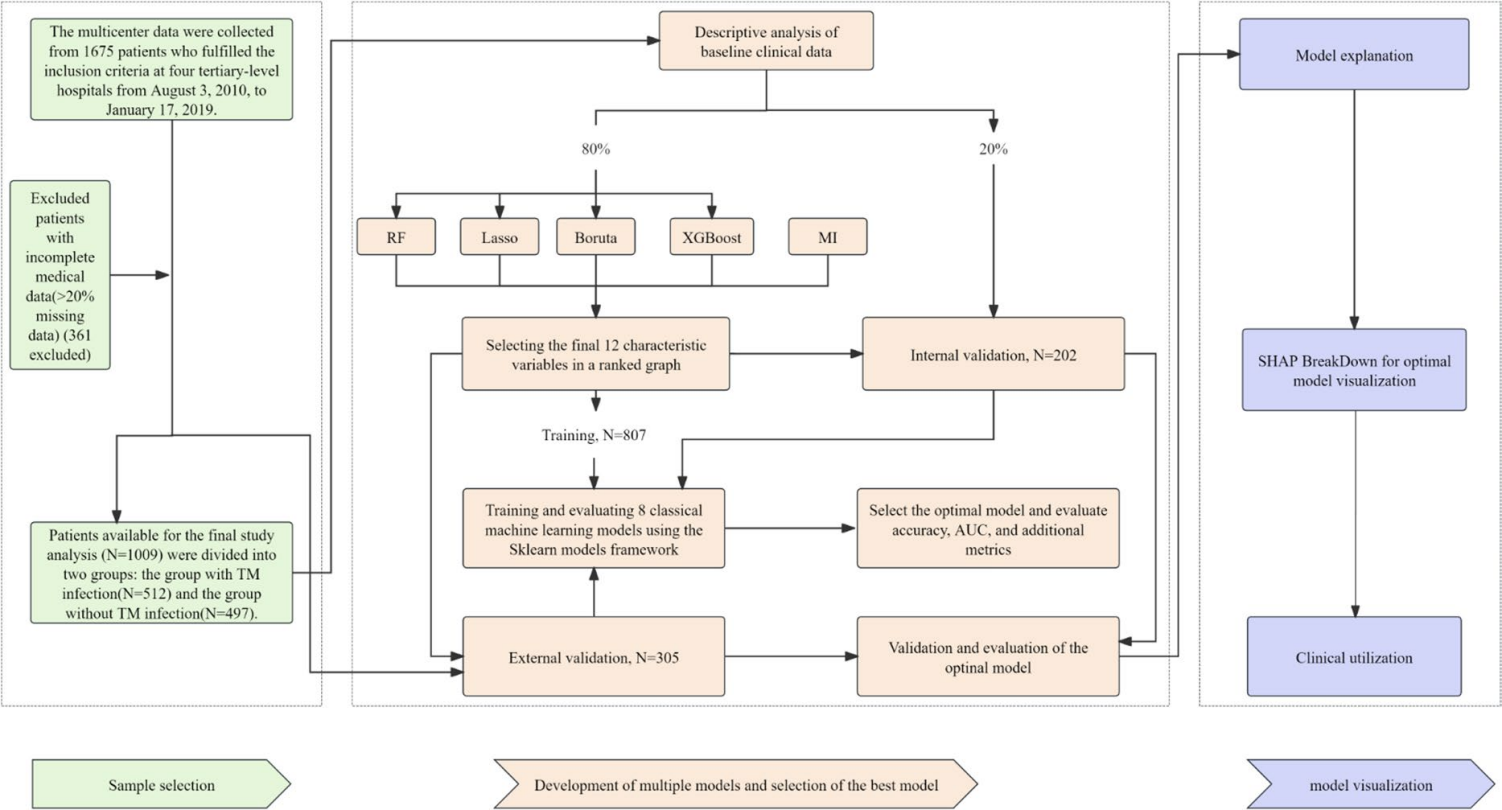
Flow chart for model development and validation

In this retrospective study, an initial screening was conducted on 1675 hospitalized patients diagnosed with HIV who met the inclusion criteria. Following this, 361 patients (21.5%) were excluded by predefined criteria, as they had missing data exceeding 20% of the total variables. The modeling cohort, comprising 1,009 patients, was randomly divided into training and testing subsets with an 80:20 ratio to enable internal validation. Furthermore, a distinct cohort of 305 patients was used for external validation. For a detailed illustration of the study’s structure, please refer to Fig. 1. Furthermore, within the modeling group, patients were categorized into two subgroups based on the presence of talaromycosis, as shown in Table 1. Significant differences (*p* < 0.05) were observed between the talaromycosis group (n = 512) and the non-talaromycosis group (n = 497) in age, BMI, fever, poor appetite, POAL, hepatomegaly, splenomegaly, AST, ALT, AST/ALT ratio, ALB, Hb, WBC count, ALC, CRP, PLT count, and CD4^+^ T-cell count levels. Supplementary Figure S1 demonstrates the correlation between variables, while Supplementary Figure S2 depicts the distribution of 36 independent variables.

**Table 1:** Baseline characteristics in the modeling group

Predictive Variables	Levels	Total (n = 1009)	Without TM infection (n = 497)	With TM infection (n = 512)	*P*
Sex, N (%)	Male	747(74.1%)	372 (74.8%)	375 (73.2%)	0.561
	Female	262(25.9%)	125 (25.2%)	137 (26.8%)	
Age (years)	median (IQR)	51.0(40.0–64.0)	55.0(44.0–68.0)	48.0(38.3–59.0)	< 0.001
BMI (kg/m^2^)	median (IQR)	19.4(18.0–20.0)	19.4(18.0–20.2)	19.3(18.0–20.0)	< 0.001
Nationality(Han), N (%)	No	219(21.7%)	101 (20.3%)	118 (23.0%)	0.294
	Yes	790(78.3%)	396 (79.7%)	394 (77.0%)	
Occupation, N (%)	Farmer	400(39.6%)	194(39.0%)	206(40.2%)	0.091
	Unemployed	156(15.5%)	66(13.3%)	90(17.6%)	
	Other	453(44.9%)	237(47.7%)	216(42.2%)	
Marital status, N (%)	Married	718(71.1%)	354(71.2%)	364(71.1%)	0.233
	Single	143(14.2%)	63(12.7%)	80(15.6%)	
	Other	148(14.7%)	80(16.10)	68(13.3%)	
Injection drug user, N (%)	No	976(96.8%)	481(96.8%)	495(96.7%)	0.928
	Yes	33(3.2%)	16(3.2%)	17(3.3%)	
Oral candidiasis, N (%)	No	402(39.9%)	198(39.8%)	204(39.8%)	0.999
	Yes	607(60.1%)	299(60.2%)	308(60.2%)	
Tuberculosis, N (%)	No	852(84.4%)	422(85.0%)	430(84.0%)	0.685
	Yes	157(15.6%)	75(15.0%)	82(16.0%)	
Bacterial Pneumonia, N (%)	No	710(70.4%)	352(70.8%)	358(69.9%)	0.753
	Yes	299(29.6%)	145(29.2%)	154(30.1%)	
Pneumocystis pneumonia, N (%)	No	768(76.1%)	378(76.0%)	390(76.2%)	0.966
	Yes	241(23.9%)	119(24.0%)	122(23.8%)	
Cryptococcosis, N (%)	No	988(97.9%)	486(97.8%)	502(98.0%)	0.772
	Yes	21(2.1%)	11(2.2%)	10(2.0%)	
Cytomegalovirus disease, N (%)	No	923(91.5%)	452(91.0%)	471(92.0%)	0.552
	Yes	86(8.5%)	45(9.0%)	41(8.0%)	
Herpes simplex virus disease,N (%)	No	961(95.2%)	475(95.6%)	486(94.9%)	0.627
	Yes	48(4.8%)	22(4.4%)	26(5.1%)	
Lymphoma, N (%)	No	996(99.0%)	490(98.6%)	506(98.8%)	0.739
	Yes	13(1.0%)	7(1.4%)	6(1.2%)	
Hepatitis B, N (%)	No	911(90.3%)	453(91.1%)	458(89.5%)	0.364
	Yes	98(9.7%)	44(8.9%)	54(10.5%)	
Hepatitis C, N (%)	No	979(97.0%)	485(97.6%)	494(96.5%)	0.303
	Yes	30(3.0%)	12(2.4%)	18(3.5%)	
Fever, N (%)	No	482(47.7%)	291(58.5%)	191(37.3%)	< 0.001
	Yes	527(52.2%)	206(41.4%)	321(62.7%)	
Cough, N (%)	No	478(47.3%)	223(44.8%)	255(49.8%)	0.116
	Yes	531(52.6%)	274(55.1%)	257(50.2%)	
Poor appetite, N (%)	No	691(68.4%)	401(80.6%)	290(56.6%)	< 0.001
	Yes	318(31.5%)	96(19.3%)	222(43.4%%)	
POAL, N (%)	No	700(69.3%)	433(87.1%)	267(52.2%)	< 0.001
	Yes	309(30.6%)	64(12.8%)	245(47.8%)	
Hepatomegaly, N (%)	No	885(87.7%)	478(96.1%)	407(79.5%)	< 0.001
	Yes	124(12.2%)	19(3.8%)	105(20.5%)	
Splenomegaly, N (%)	No	886(87.8%)	472(94.9%)	414(80.9%)	< 0.001
	Yes	123(12.1%)	25(5.0%)	98(19.1%)	
Hb (g/L)	median (IQR)	100.0(84.0–114.0)	105.0(92.0–119.5)	93.0(78.0–108.0)	< 0.001
WBC (× 10^9^/L)	median (IQR)	4.6(3.2–6.7)	5.2(3.9–7.2)	4.1(2.8–6.2)	< 0.001
ALC(× 10^9^/L)	median (IQR)	0.6(0.3–0.9)	0.7(0.5–1.1)	0.4(0.3–0.7)	< 0.001
CRP(mg/L)	median (IQR)	47.9(17.5–69.6)	34.7(11.0–61.5)	62.0(29.4–75.0)	< 0.001
PLT (× 10^9^/L)	median (IQR)	188.0(110.0–257.5)	216.0(158.5–284.5)	143.0(74.0–220.0)	< 0.001
AST(U/L)	median (IQR)	43.0(26.0–78.0)	31.0(22.0–47.0)	62.5(35.8–110.5)	< 0.001
ALT(U/L)	median (IQR)	26.0(16.0–46.0)	22.0(14.0–35.4)	34.0(20.0–59.0)	< 0.001
AST/ALT	median (IQR)	1.7(1.1–2.5)	1.5(1.1–2.1)	1.9(1.3–3.0)	< 0.001
ALB(g/L)	median (IQR)	28.7(24.2–32.6)	30.5(26.6–34.3)	26.1(22.6–30.1)	< 0.001
BUN (mmol/L)	median (IQR)	4.6(3.4–6.3)	4.7(3.4–6.3)	4.4(3.3–6.3)	0.357
Creatinine (mg/L)	median (IQR)	70.9(58.0–85.3)	69.9(59.3–86.2)	71.0(57.0–85.0)	0.885
G(pg/mL)	median (IQR)	190.0(38.5–255.0)	182.0(35.0–251.5)	202.0(43.0–256.8)	0.651
CD4 + T cell count (cells/μL)	median (IQR)	20.0(9.0–43.5)	28.0(13.0–58.5)	14.0(6.3–29.0)	< 0.001

Abbreviations: BMI, Body mass index; POAL, Peripheral or abdominal lymphadenopathy; HB, Hemoglobin; WBC, White blood cell; ALC, Absolute lymphocyte count; CRP, C-reactive protein; PLT, Platelet; AST, Aspartate aminotransferase; ALT, Alanine transaminase; ALB, albumin; BUN, Blood urea nitrogen; G, (1–3)-β-D glucan; IQR, Interquartile range

### Variable Screening

**Fig. 2:**
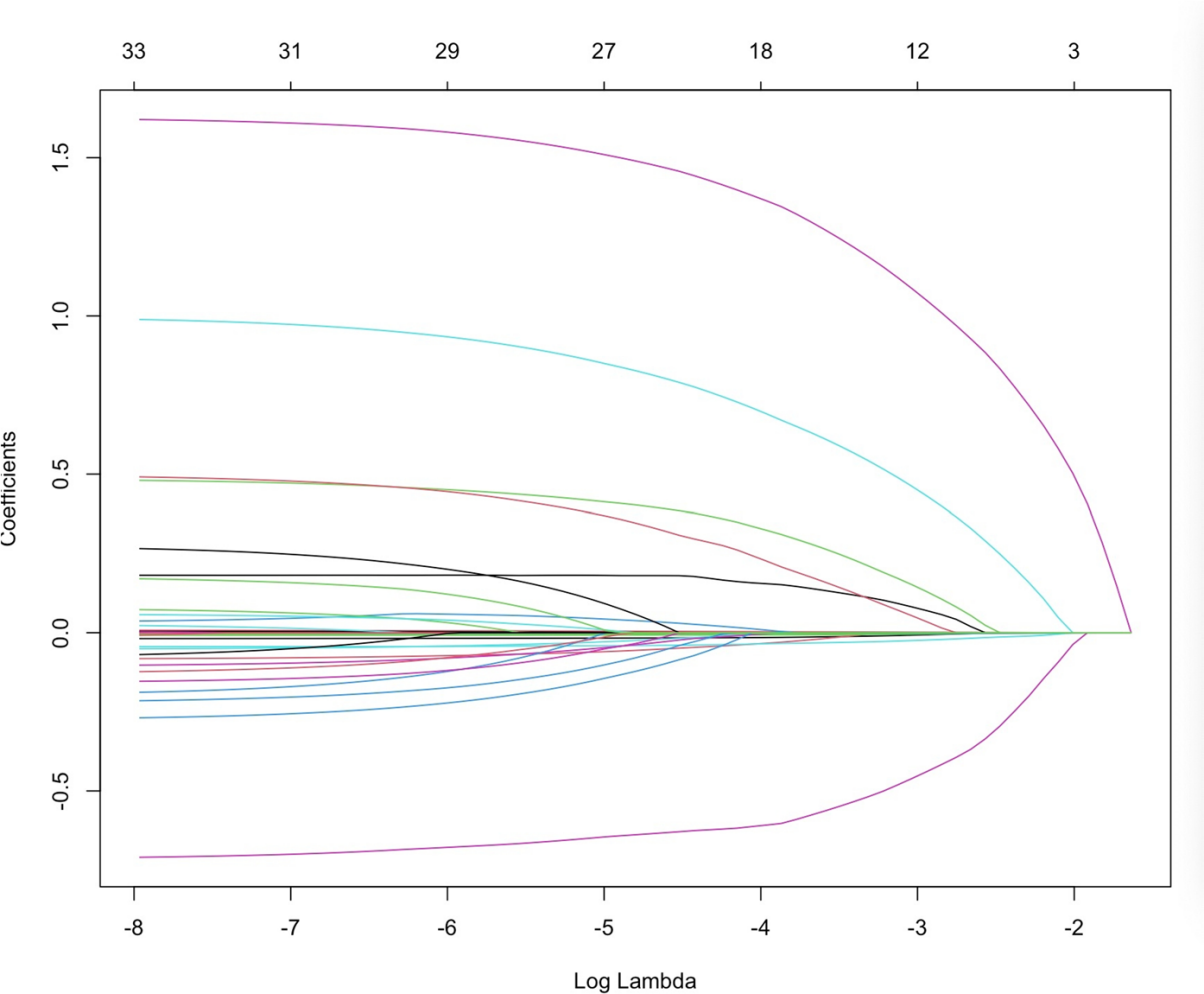
The screening process of predictive variables(coefficient distribution). In LASSO regression, coefficients shrink as the regularization strength (log Lambda) increases, with non-essential variables nearing zero

**Fig. 3:**
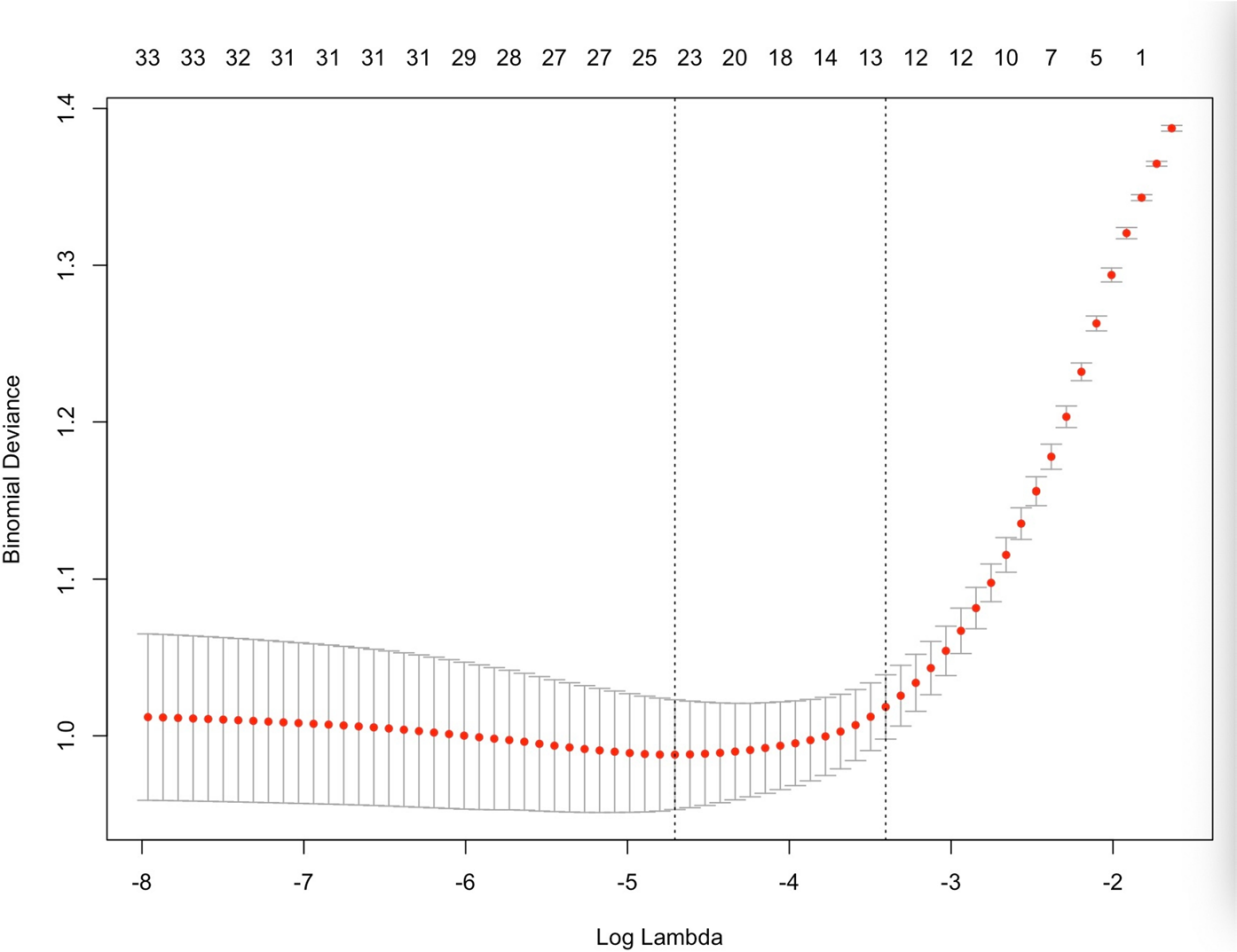
The screening process of predictive variables(cross-validation). The coefficient paths show how predictor shrinkage occurs with increasing regularization strength (log Lambda). The top numbers (33 to 3) indicate retained variables, and coefficients (+ 1.0 to 8) represent their weights. As regularization strengthens (higher log Lambda), non-essential variables are reduced to zero, allowing for automated feature selection

**Fig. 4:**
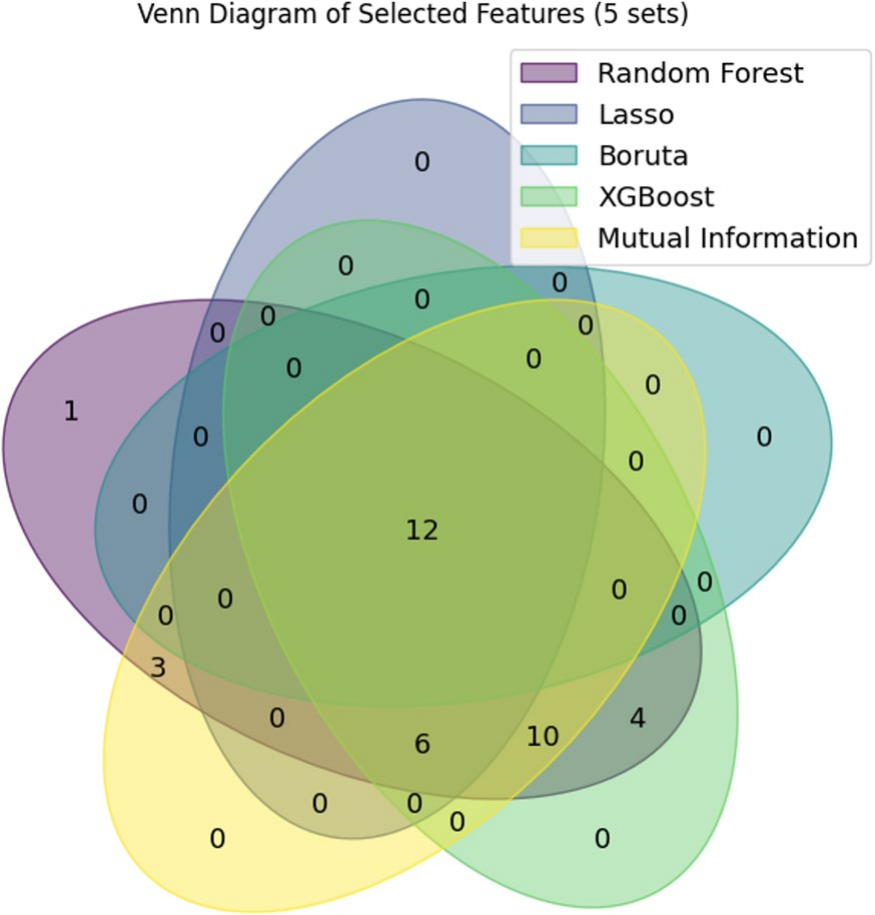
Variable Wayne diagram screened by five methods. This Venn diagram shows the overlap of features chosen by five feature selection methods: Random Forest, Lasso, Boruta, XGBoost, and Mutual Information. Each section represents features chosen by a specific method, with overlaps indicating features selected by multiple methods. The center highlights 12 features identified by all methods, emphasizing their significance in model building

Five complementary feature selection methods-RF, LR, Boruta, XGBoost, and MI-were applied to the 36 candidate variables. Detailed results from each method, including variable importance rankings and coefficient trajectories, are provided in Figs. 2–3, Supplementary Figs. S3–S9, and Supplementary Table S1. The intersection of variables selected by all five methods yielded 12 common predictors: ALB, ALC, HB, AST/ALT ratio, CRP, ALT, WBC, PLT, POAL, CD4, AST, and age. A Venn diagram illustrating the overlap is shown in Fig. 4. The comprehensive procedure for variable screening is provided in Supplementary Methods 2.

### Development and Evaluation of the Model

Utilizing the 12 selected variables, we developed eight machine learning models: LR, SVM, RF, MLP, NB, DT, KNN, and XGBoost. The performance of these models was evaluated through tenfold cross-validation and assessed using metrics such as the AUC, ACC, sensitivity, specificity, and F1-score. A detailed comparison of all models is provided in Supplementary Table S2. Among the eight models, the SVM exhibited the superior and most consistent performance across training, testing, and external validation cohorts. In external validation, the SVM achieved an AUC of 0.921 (95% CI 0.889–0.951), an ACC of 0.853, a sensitivity of 0.823, and a specificity of 0.873. Other models, including LR and NB, also demonstrated strong performance, albeit with slightly lower metrics. Models such as DT, RF, and XGBoost displayed indications of overfitting, achieving perfect training performance but experiencing declines during validation. Consequently, the SVM was selected as the final model due to its robustness and generalizability.

### Validation of the Final Model was Conducted both Internally and Externally

**Fig. 5:**
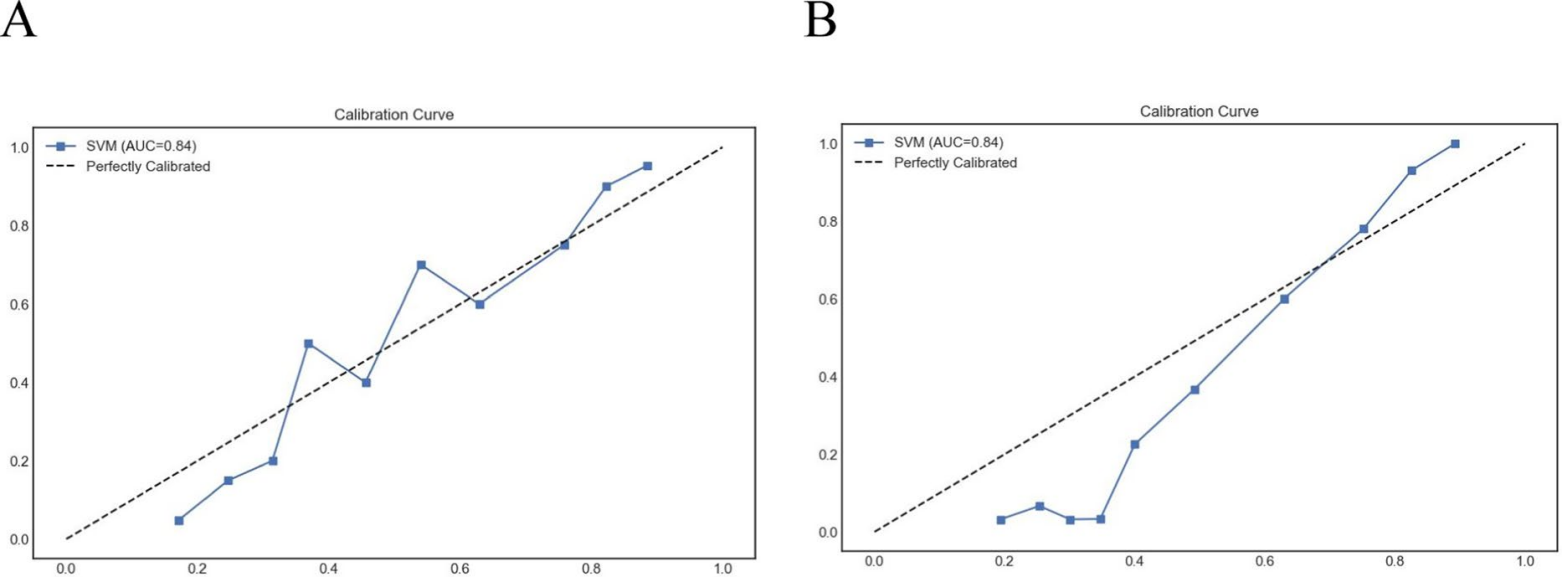
The SVM model’s calibration curve was evaluated using the test dataset and external validation. **A** Calibration curve of the SVM model evaluated on the test dataset. **B** Calibration curve of the SVM model assessed during external validation. Calibration curves for the Support Vector Machine (SVM) model (AUC = 0.84) in predicting Talaromyces marneffei infection are presented. The curves for both the test dataset **A** and the external validation cohort **B** demonstrate alignment with the ideal calibration line, thereby confirming the reliability of the predicted probabilities across different cohorts

**Fig. 6:**
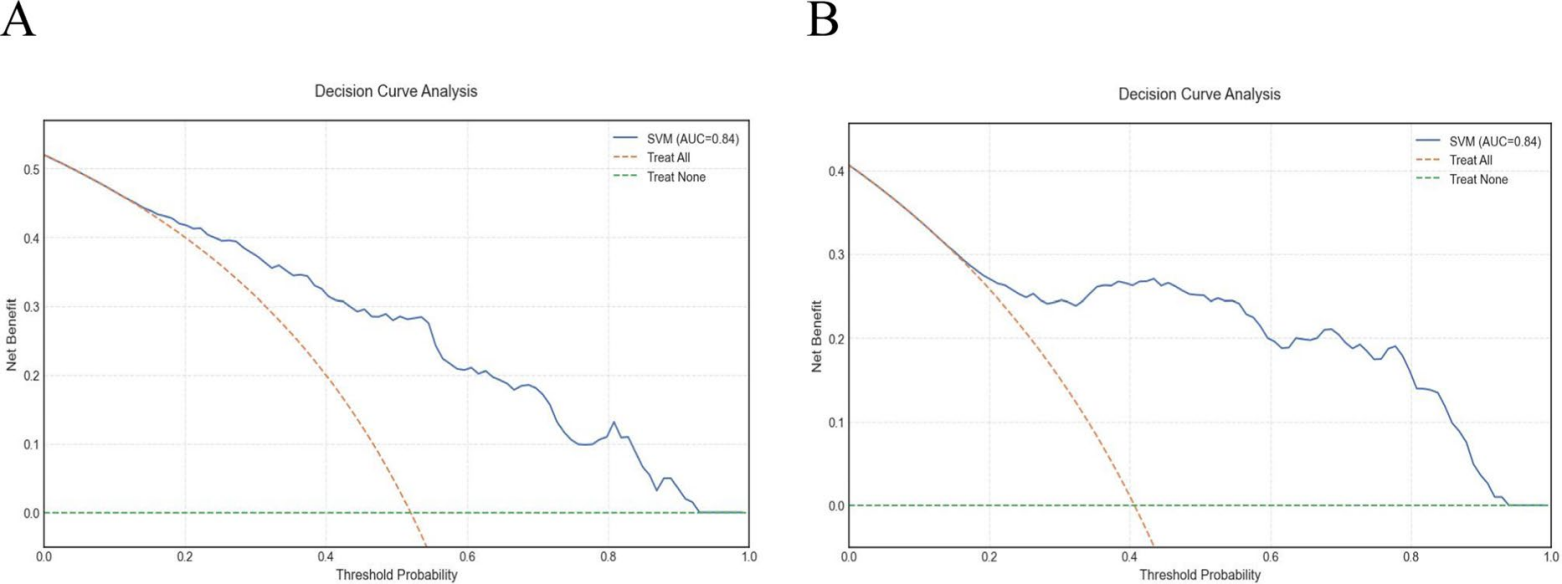
The SVM model’s DCA was evaluated using both the test dataset and external validation. **A** The DCA of the SVM model was evaluated on the test dataset.** B** The DCA of the SVM model was assessed during external validation. Decision curve analysis of the SVM model (AUC = 0.84) for predicting Talaromyces marneffei infection shows that both the test and external validation datasets yield a higher net benefit than “Treat All” or “Treat None” strategies across threshold probabilities (0.1–0.5 vs. 0.2–1.0), indicating clinical usefulness

The reliability and clinical utility of the SVM model were rigorously validated using calibration curves and DCA across both internal and external cohorts. Internally, the calibration curve (Fig. 5A) exhibited near-perfect alignment with the ideal line (AUC = 0.84), thereby confirming accurate risk estimation. The DCA (Fig. 6A) further demonstrated a superior net benefit compared to default strategies, particularly at thresholds ranging from 10 to 50%, supporting its application for prioritizing high-risk patients. Externally, the model maintained robust calibration (Fig. 5 B) with minimal deviation in high-risk ranges and sustained a positive net benefit (Fig. 6 B) across thresholds from 20 to 100%. These findings underscore the model’s generalizability, as it consistently balanced sensitivity and specificity while minimizing unnecessary interventions. The concordance between internal and external validation highlights its readiness for clinical implementation in diverse settings, providing a reliable tool for the early detection of *Talaromyces marneffei* infection in HIV patients.

### Analysis of the Model’s Implications

**Fig. 7:**
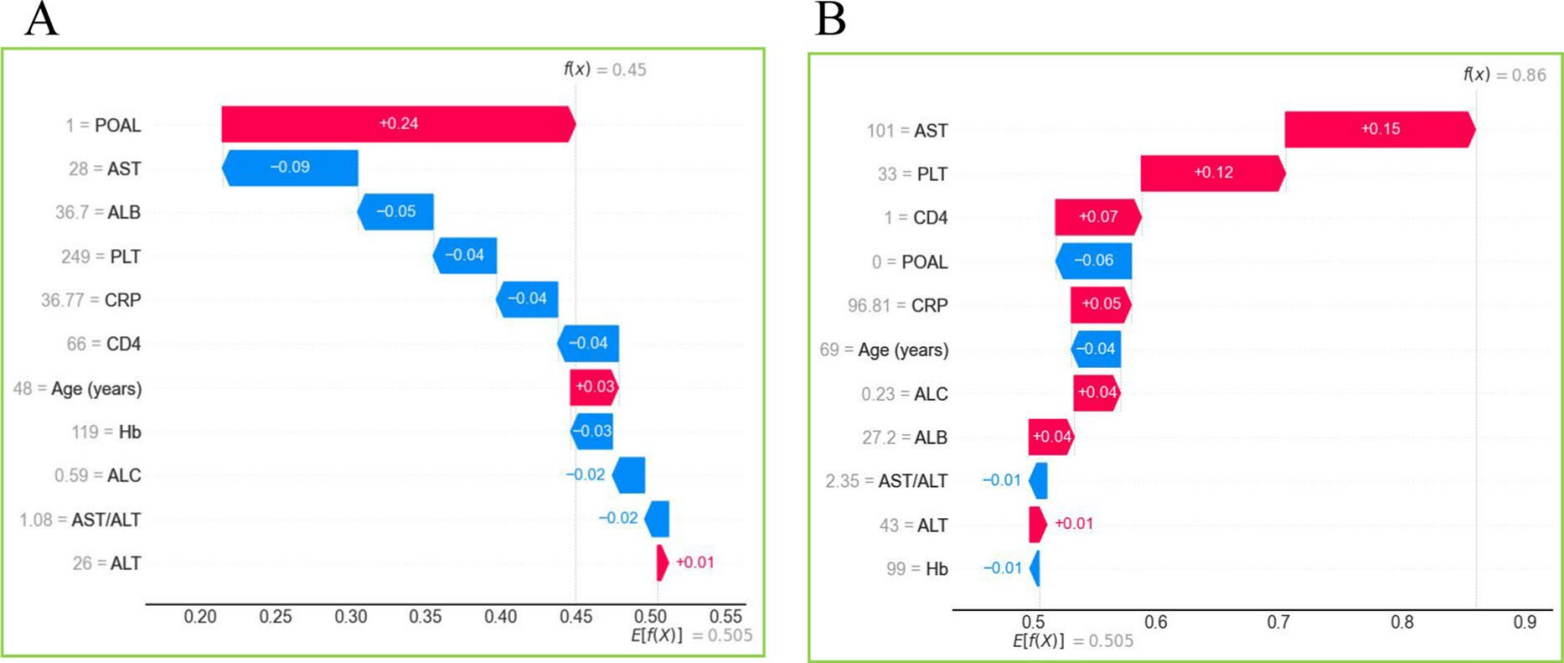
Directional SHAP contributions in predicting *Talaromyces marneffei* infection. **A** Positivedrivers: Elevated AST (101), CRP (96.8), and Hb (99) strongly increase risk (SHAP > 0). **B** Negative drivers: POAL (0) and AST/ALT (2.35) reduce risk (SHAP < 0), while ALT (43) and AST (28) show context-dependent effects. SHAP magnitude reflects impact relative to baseline prediction E[f(X)] = 0.505E[f(X)] = 0.505

The SHAP framework offers a visualization tool tailored for clinicians, aimed at clarifying the influence of specific clinical features on predictions of active infection. As illustrated in Supplementary Fig. S10, the selected set of 12 features demonstrates differential impacts on the prediction of TM infection. Among these, POAL, AST, and ALC are particularly noteworthy due to their significant contributions to the model’s predictive accuracy, thereby establishing them as essential diagnostic markers. Figure 7A illustrates a high-risk patient (f(x) = 0.86), whose prediction is primarily influenced by elevated AST levels (101 U/L, with a normal range of 7–40 U/L) and severe thrombocytopenia (platelet count = 33 × 10^9^/L, with a normal range of 100–300 × 10^9^/L), contributing SHAP values of + 0.15 and + 0.12, respectively. These biomarkers are consistent with established pathophysiological mechanisms, as hepatic injury and bone marrow suppression are characteristic features of disseminated *Talaromyces marneffei* infection in immunocompromised individuals. In contrast, Fig. 7B depicts a low-risk scenario (f(x) = 0.45), where protective factors such as normal platelet counts (PLT = 249 × 10^9^/L, SHAP − 0.04) and elevated albumin levels (ALB = 36.7 g/L, within the normal range of 35–55 g/L, SHAP − 0.05) reduce the likelihood of infection. Both predictions are contextualized against the baseline risk (E[f(X)] = 0.505), which represents the average infection probability across the cohort.

### Development of User-Friendly Applications

Our predictive model can be used locally or accessed online via Gradio, a platform that enables application sharing without requiring Python code. The application computes the probability of talaromycosis in people living with HIV without skin lesions using the specific values of the 12 predictor variables, as shown in Supplementary Fig. S11A and B. The web application is available to users in China (https://modelscope.cn/studios/LRYHJG/rf/summary) and internationally (https://huggingface.co/spaces/HuJiaGuang/LRYHJG-TM).

## Discussion

This multicenter retrospective cohort study utilized EMR data from both endemic and non-endemic regions in China to develop an interpretable ML model aimed at predicting the presence of talaromycosis specifically in people living with HIV without skin lesions. The SVM -based model exhibited a strong discriminative capacity, achieving an AUC of 0.921 in external validation, and demonstrated significant clinical applicability, as confirmed by DCA. This study is distinguished from previous talaromycosis diagnostic prediction research in three key aspects: firstly, its explicit focus on patients without skin lesions, a demographic that has not been previously reported in the literature; secondly, the employment of a rigorous multi-algorithm feature selection strategy, which intersects results from RF, Lasso, XGBoost, Boruta, and MI, thereby enhancing the model’s stability and generalizability; and thirdly, the incorporation of SHAP-based interpretability alongside a fully deployed web-based tool, effectively bridging the gap between complex ML methodologies and bedside clinical utility. This comprehensive framework represents a significant advancement over previously published models, which have either predominantly relied on skin lesions as a primary predictor or lacked accessibility[18].

It is commonly recognized that ML models provide enhanced predictive capabilities compared to conventional models in the medical field [26]. This benefit facilitates the development of a more robust model from complex datasets [27]. This study underscores the proficiency of ML in managing heterogeneous multidimensional data through the integration of twelve predictive variables. For example, the elevation of AST and the reduction of PLT show high heterogeneity. Additionally, we employed visualization techniques to clarify the forecast model and create functional web applications. This step is crucial for addressing concerns related to the opacity of black-box models and for rebuilding trust in ML within the medical sector [28]. To enhance reproducibility, the model development process incorporated cross-checking, self-validation, and outside confirmation, all conducted with predetermined random seeds. This is a crucial step to ensure the clinical significance of the model through its interpretability [29].

In recent years, research into TM infection among hospitalized people living with HIV has increased. This infection is predominantly observed in Southeast Asia, particularly among immunocompromised individuals. Although numerous studies have concentrated on the prognostic risks associated with these patients, there is a relative paucity of research dedicated to predicting the diagnosis of infection. Xu L et al. [18] employed multivariable logistic regression to develop a predictive model based on clinical indicators for ten key factors and assessed the risk of talaromycosis in hospitalized people living with HIV. The model achieved AUC values of 0.883 in the training set and 0.889 in the validation set, underscoring the significant role of skin lesions as a critical variable in disease prediction within diagnostic models. For the approximately one-third of TM patients who do not exhibit skin lesions [11], depending solely on this clinical feature could result in delayed diagnosis or missed opportunities for initiating early antifungal treatment. This study specifically targets this clinical gap by developing a model tailored for this subgroup, thereby enhancing and not simply duplicating existing research. Moreover, unlike Xu L et al. [18], who utilized a single-model nomogram approach, our methodology employs a multi-algorithm feature selection combined with an SVM framework. This approach is capable of capturing complex non-linear interactions among predictors, potentially providing superior discrimination for this challenging patient subgroup.

To substantiate the increased complexity of our ML methodology, we conducted a direct performance comparison with more traditional statistical models. Among the eight algorithms assessed, LR was selected as a representative of conventional clinical risk modeling. During external validation, the SVM model (AUC 0.921, ACC 0.853) exhibited a significant performance enhancement over LR (AUC 0.892, ACC 0.823), as detailed in Supplementary Table S2. Although LR provides inherent interpretability, its sensitivity (0.795) was markedly lower than that of the SVM (0.823). In the context of a life-threatening opportunistic infection, this increase in sensitivity corresponds to approximately three fewer missed diagnoses per 100 high-risk patients, which is a clinically significant margin. Furthermore, the operational complexity of an advanced ML model is effectively mitigated by our web-based deployment, which automates all calculations and delivers results via an intuitive interface. Consequently, the amalgamation of enhanced predictive performance, SHAP-based interpretability, and automated deployment substantiates the adoption of a more sophisticated modeling approach over simpler alternatives.

We created and externally validated a talaromycosis diagnostic prediction model for people living with HIV who lack skin lesions, using 12 clinical variables from the electronic medical records of four hospitals. To address collinearity in the model development data, we used RF, Lasso, XGBoost, Boruta, and MI to select the 36 influential variables, employing L1 regularization. The study aimed to create a practical clinical application by selecting a limited number of readily accessible patient markers. Taking practicality into account, if a single marker can fulfill the prediction objective, there is no need to incorporate additional variables. More significantly, these predictive variables serve as baseline markers. Additionally, drawing on the findings from previous literature, these 36 variables are frequently used as indicators in similar research studies. The study retrospectively analyzed 9 years of patient data. Moreover, these 36 variables represent the most extensive and readily accessible data within our electronic medical records system. We employed eight ML algorithms to individually fit each of the 12 predictive variables, aiming to achieve optimal fitting results. The SVM algorithm generated a model with a high ACC (0.714) and an AUC of 0.809. This model performed exceptionally well on both internal and external validation datasets.

Xu L et al. [18] reported that the AST, PLT, age, POAL, and CD4^+^ T-cell count were independent factors influencing the diagnosis of TM infection and subsequently formulated a clinically applicable predictive model based on these findings. Our study has arrived at similar conclusions, with these five variables standing out as key contributors to the diagnosis of TM infection. This finding is further corroborated by the network application we developed using Gradio.

Studies indicate that TM infections are more prevalent and deadly among individuals infected with HIV in Southeast Asia [1]. Upon diagnosis, immediate and active treatment measures must be taken. High model ACC is essential to reduce false negatives (missed diagnoses) and false positives (misdiagnoses). The SVM model demonstrated robust diagnostic performance during the validation process. During the training phase, it achieved an ACC of 0.714, an AUC of 0.809, a specificity of 0.795, and a sensitivity of 0.631. Notably, the model’s performance metrics, including ACC, AUC, specificity, sensitivity, and F1-score, showed improvement in both the testing and external validation datasets compared to the training phase. For instance, in the external validation cohort, the SVM model attained an AUC of 0.921, an ACC of 0.853, and an F1-score of 0.819. These enhancements underscore the model’s strong generalization capability and reliability. The model’s high accuracy, specificity, and sensitivity in detecting TM infection render it highly promising for clinical application in the early diagnosis and risk stratification of TM infection in people living with HIV who lack skin lesions. Its consistent performance across multiple validation stages further emphasizes its potential utility in real-world clinical settings. The model’s capability for risk stratification is consistent with the clinical objectives outlined in the World Health Organization’s 2023 guidelines on priority fungal pathogens [30].

Our study has several limitations that we must acknowledge. Firstly, although the model underwent external validation using an independent cohort, all data were derived from hospitals located within China. Despite the multicenter design capturing some degree of geographic diversity across Guangxi, Guangdong, and Chongqing, the model’s efficacy in other endemic regions—such as Vietnam, Thailand, India, or other areas of Southeast Asia—has not yet been determined. Geographic variations in host genetics, HIV subtype distribution, environmental exposure to *T. marneffei*, and healthcare-seeking behaviors could potentially influence predictive accuracy [31–33]. The heterogeneity among clusters, such as hospitals within China, was not quantitatively assessed due to the retrospective nature of the study and its primary emphasis on model development. Future research should prioritize international collaborations to validate and, if necessary, recalibrate the model for application in diverse global contexts. The methodological framework and the biologically grounded predictors (e.g., CD4^+^ T-cell count, liver function, platelets) offer a robust foundation for such external adaptation [34]. Second, the retrospective, hospital-based nature of the dataset necessitates several considerations. Selection bias may have been introduced due to our inclusion criteria, which required hospitalization for more than three days and a CD4^+^ T-cell count below 200 cells/μL. Although this approach targets the highest-risk population, it may restrict the generalizability of the findings to outpatient settings or people living with HIV who have a higher CD4^+^ T-cell count. Concerning class imbalance, our derivation cohort exhibited a balanced distribution (512 talaromycosis cases versus 497 non-cases), which facilitated effective model training. However, the prevalence of talaromycosis in real-world clinical settings varies significantly, ranging from 3.3% in certain Thai cohorts to 17.3% in southern China [1, 4]. Model performance metrics, particularly the positive predictive value, are sensitive to disease prevalence. In settings with lower prevalence, the model may produce a higher rate of false positives, potentially resulting in unnecessary antifungal therapy. Consequently, users of our web tool should interpret the output probability in light of their local disease epidemiology. Third, although the Gradio-based application constitutes a significant advancement toward clinical implementation, there is presently a lack of direct evidence regarding its integration into clinical workflows. Formal evaluations-such as clinician feedback surveys, think-aloud protocols during simulated use, or pilot testing in actual clinical settings-have yet to be conducted. These assessments are crucial for determining whether the tool integrates seamlessly into busy clinical environments, whether clinicians trust and accurately interpret the SHAP-based explanations, and whether its use ultimately influences clinical decision-making or patient outcomes. The current deployment is considered an essential prerequisite for conducting such studies. Prospective, multicenter implementation trials, including our ongoing trial ChiCTR1900021195, are currently in progress to systematically evaluate these aspects. These trials will assess real-time predictive accuracy, clinician acceptance, impact on time-to-diagnosis, and cost-effectiveness in routine care.

## Conclusions

To conclude, we have created a clinically beneficial and user-friendly network application. While prospective validation remains essential, predicting talaromycosis in people living with HIV without skin lesions is now possible in endemic regions. This advancement facilitates proactive and targeted clinical decision-making.

## Supplementary Information

Below is the link to the electronic supplementary material.

Supplementary file1 (ZIP 1622 KB)

## Data Availability

The dataset utilized in this research is accessible through the corresponding author upon submission of a reasonable request. The analytical code, comprising Python scripts for preprocessing, feature selection, model training, evaluation, and web application development, along with a user manual, is available in the Supplementary Materials (Supplementary Code 1, Supplementary File 1 – User Manual, Supplementary File 2 – Web-based Diagnostic Prediction Tool Development Process, and Supplementary File 3-Web Diagnostic Prediction Tool Material Package). The data from the study can be obtained from the corresponding author upon a reasonable request.
