# Supplementary material for: An Interpretable Machine Learning Model for Predicting the Presence of Talaromycosis in HIV Patients Lacking Skin Lesions
Source: Mycopathologia. 2026 Jul 21;191(4):66. doi: 10.1007/s11046-026-01089-y (PMC13384986; doi:10.1007/s11046-026-01089-y)
Supplement: Supplementary file 1 — Supplementary file1 (ZIP 1622 KB) [file 11046_2026_1089_MOESM1_ESM.zip › ESM/fig legend.docx]

**Legends for Supplementary Figures**

**Supplementary Figure 1. Correlation Heatmap of Feature in the training cohorts**

Color intensity indicates pairwise Pearson coefficients (-1 to +1); red signifies positive and blue negative. Key biomarkers like CRP and AST moderately correlate with infection-related variables such as bacterial pneumonia and cytomegalovirus.

**Supplementary Fig. S2.  Distribution of 36 independent variables in the training cohort**

Scatter plots illustrate the distribution of specific features (e.g., Oral candidiasis, Lymphoma, CRP, and AST) with sample index on the X-axis and normalized values on the Y-axis, showcasing the variability and heterogeneity in clinical biomarkers.

**Supplementary Fig. S3. Relationship between tree number and error rates in Random Forest model for Talaromyces marneffei infection prediction.**As the number of trees increases, the error rates for both the infected (represented in green) and non-infected (represented in blue) groups decrease, eventually converging with the out-of-bag (OOB) error rate (represented in magenta) beyond approximately 150 trees. This convergence suggests a stabilization in model performance.

**Supplementary Fig. S4. List 36 predictive variables in descending order of importance**

**Supplementary Fig. S5. 12 Non-zero predictive variables selected by Lasso regression screening**

LASSO identified key predictors for diagnosing Talaromyces marneffei infection, with twelve features retained as significant clinical biomarkers.

**Supplementary Fig. S6. Feature importance with confidence intervals (Boruta)**

Boruta-selected key biomarkers (e.g., ALT, CRP, Age) for diagnosing Talaromyces marneffei infection are ranked by Random Forest importance scores, with error bars indicating model stability. This ranking highlights statistically significant predictors.

**Supplementary Fig. S7**. **Feature importance ranking**

The evolution of feature importance ranking through Boruta iterations shows initial high variability as the model assesses feature significance (lower ranks indicate higher importance). Later stabilization indicates convergence on key predictors of Talaromyces marneffei infection.

**Supplementary Fig. S8. The variables selected by XGBoost are sorted by importance**

XGBoost-ranked feature importance for predicting Talaromyces marneffei infection highlights key predictors like AST, CRP, and POAL based on their importance scores, showing their contribution to the model. Non-zero weights indicate biomarkers retained after regularization.

**Supplementary Fig. S9. The variables selected by MI are sorted by importance**

Biomarkers ranked by mutual information for predicting Talaromyces marneffei infection show key features like AST, PLT, and ALB, grouped by clinical relevance, such as splenomegaly and bacterial pneumonia, with higher MI scores indicating stronger outcome dependencies.

**Supplementary Fig. S10. Mean absolute SHAP values rank feature importance for predicting Talaromyces marneffei infection**

Key predictors like POAL, AST, and ALC show their magnitude of impact on the model through bar length.

**Supplementary Figure 11. A web-based SVM model was developed to predict talaromycosis**

By entering a participant's clinical variables into the online tool available at https://modelscope.cn/studios/LRYHJG/rf/summary for users in China and https://huggingface.co/spaces/HuJiaGuang/LRYHJG-TM for international users, the corresponding probability of developing talaromycosis can be calculated. As demonstrated in Figures A and B, the probabilities of talaromycosis development for the two patients in our study were determined to be 0.64 and 0.84, respectively.

**Supplementary Fig. S12.** DeLong test *P*-values comparing each model's performance

to the top model in the test group.

The top model (SVM, *P*=1.00) was used as the benchmark. A *P*-value below 0.05 signifies a statistically significant performance difference (e.g., AUC) from this model.
