# Supplementary material for: An Interpretable Machine Learning Model for Predicting the Presence of Talaromycosis in HIV Patients Lacking Skin Lesions
Source: Mycopathologia. 2026 Jul 21;191(4):66. doi: 10.1007/s11046-026-01089-y (PMC13384986; doi:10.1007/s11046-026-01089-y)
Supplement: Supplementary file 1 — Supplementary file1 (ZIP 1622 KB) [file 11046_2026_1089_MOESM1_ESM.zip › ESM/Supplementary Fig S11 A web based SVM modelï1⁄4ˆreversionï1⁄4‰.pdf]

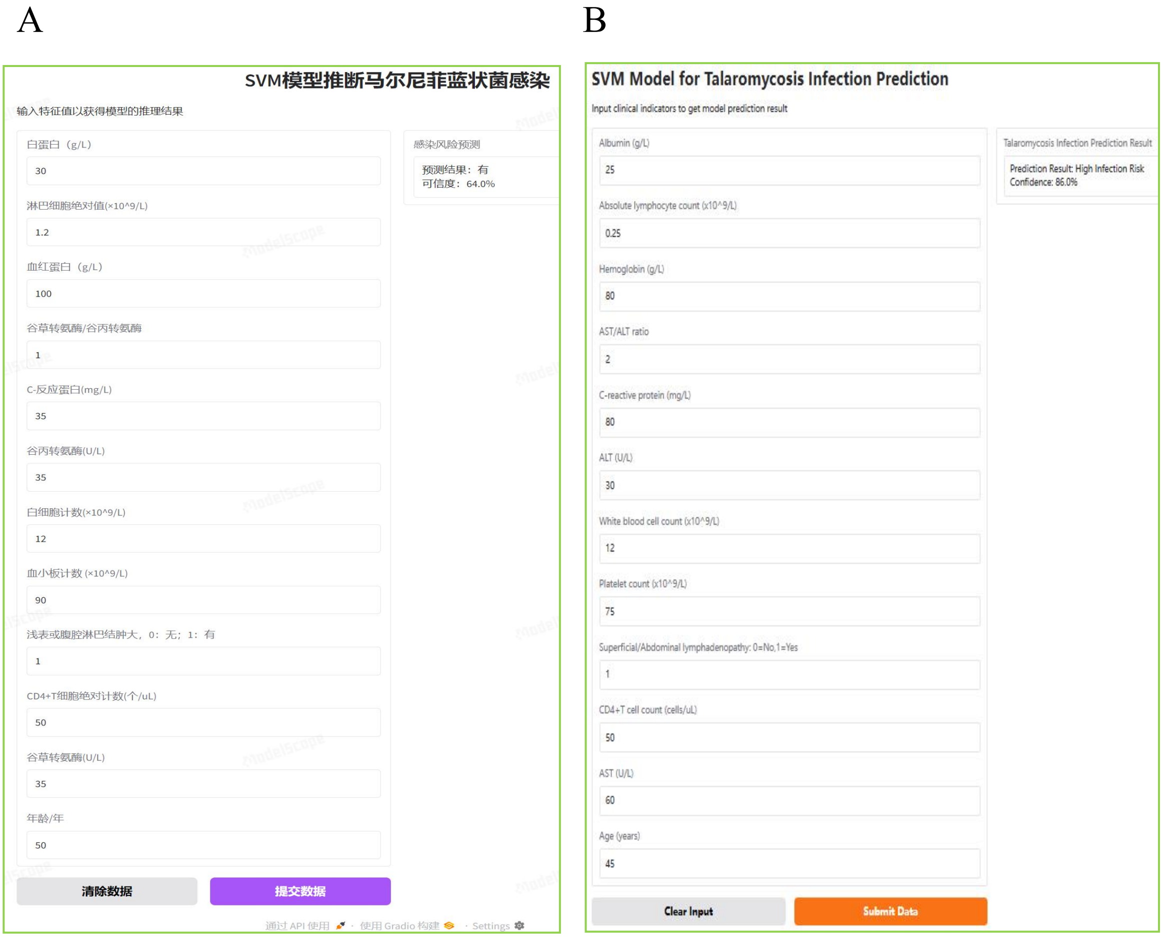

Figure 9. A web-based SVM model was developed for the prediction of talaromycosis

By entering a participant's clinical variables into the online tool available at https://modelscope.cn/studios/LRYHJG/rf/summary for users in China and https://huggingface.co/spaces/HuJiaGuang/LRYHJG-TM for international users, the corresponding probability of developing talaromycosis can be calculated. As demonstrated in Figures A and B, the probabilities of talaromycosis development for the two patients in our study were determined to be 0.64 and 0.86, respectively. The "Prediction Result" output generates a binary value of either "High Infection Risk" or "No Infection," signifying the presence or absence of a concurrent Talaromyces marneffei infection, respectively. Concurrently, the "Confidence" output value quantifies the probability associated with this likelihood. Abbreviations: ALT, Alanine transaminase; AST, Aspartate aminotransferase(U/L).
