# Supplementary material for: An Interpretable Machine Learning Model for Predicting the Presence of Talaromycosis in HIV Patients Lacking Skin Lesions
Source: Mycopathologia. 2026 Jul 21;191(4):66. doi: 10.1007/s11046-026-01089-y (PMC13384986; doi:10.1007/s11046-026-01089-y)
Supplement: Supplementary file 1 — Supplementary file1 (ZIP 1622 KB) [file 11046_2026_1089_MOESM1_ESM.zip › ESM/Supplementary Methods 1.docx]

**Feature Selection Based on Multiple Machine Learning Algorithms**

In this study, potential variables were subjected to an additional screening process based on dimensionality reduction criteria to evaluate their appropriateness for inclusion in the model [1]. The Random Forest (RF) algorithm is an ensemble method that combines several decision trees. It generates models by applying random sampling to the training dataset and determining optimal split points. Individual decision trees in RF are constructed based on feature metrics derived from dataset attributes [2, 3], facilitating a robust assessment of each feature's significance [4]. Lasso regression performs variable selection and controls model complexity through regularization, effectively preventing overfitting. The parameter λ controls regularization strength, leading to a simplified model with fewer variables. The optimal value of λ(λ_-_min) is identified using 10-fold cross-validation, where the point of minimum error is used to select the most relevant predictive variables [5, 6]. Unlike traditional feature selection methods, Borua uses a wrapper-based method to select features. It aims to determine the feature set that demonstrates the strongest association with the dependent variable, prioritizing comprehensive relevance over the creation of a minimal, model-specific subset [7]. Through the iterative elimination of low-correlation features, this method significantly reduces noise and enhances the consistency of classification outcomes [8]. Meanwhile, as a highly potent ensemble learning technique, Extreme Gradient Boosting (XGBoost) is established based on classification trees. By integrating multiple weak learners into a robust ensemble model through sequential boosting steps, it constructs a tree-based classifier, providing a reliable approach for precise classification tasks [9]. Finally, Mutual Information (MI) effectively captures non-linear relationships, complementing RF for robust feature importance, Lasso for sparsity, Boruta for comprehensive relevance, and XGBoost for high-precision ensemble learning in medical data analysis [10].

Specifically, we utilized Lasso regression, RF, Boruta, XGBoost, and MI to analyze the 36 potential risk factors. Variables with non-zero coefficients were ranked according to their contribution to the outcome, and common variables were identified by intersecting the results from all five methods.

**References**

[1] VENKATESH K K, JELOVSEK J E, HOFFMAN M, et al. Postpartum readmission for hypertension and pre‐eclampsia: development and validation of a predictive model [J]. BJOG: An International Journal of Obstetrics & Gynaecology, 2023, 130(12): 1531-40.https://doi.org/10.1111/1471-0528.17572.

[2] FARHADIAN M, TORKAMAN S, MOJARAD F. Random forest algorithm to identify factors associated with sports-related dental injuries in 6 to 13-year-old athlete children in Hamadan, Iran, 2018, a cross-sectional study [J]. BMC Sports Science, Medicine and Rehabilitation, 2020, 12(1). <https://doi.org/10.1186/s> 13102-020-00217-5.

[3] SHI G, LIU G, GAO Q, et al. A random forest algorithm-based prediction model for moderate to severe acute postoperative pain after orthopedic surgery under general anesthesia [J]. BMC Anesthesiology, 2023, 23(1). [https://doi.org/10.1186 /s12871-023-02](https://doi.org/10.1186%20/s12871-023-02) 328-1.

[4] NACHOUKI M, MOHAMED E A, MEHDI R, ABOU NAAJ M. Student course grade prediction using the random forest algorithm: Analysis of predictors' importance [J]. Trends in Neuroscience and Education, 2023, 33. <https://doi.org/10.1016/j.tine.2023>.1 00214.

[5] WANG J, XU Y, LIU L, et al. Comparison of LASSO and random forest models for predicting the risk of premature coronary artery disease [J]. BMC Medical Informatics and Decision Making, 2023, 23(1). <https://doi.org/10.1186/> s12911-023-02407-w.

[6] KANG J, CHOI Y J, KIM I-K, et al. LASSO-Based Machine Learning Algorithm for Prediction of Lymph Node Metastasis in T1 Colorectal Cancer [J]. Cancer Research and Treatment, 2021, 53(3): 773-83. <https://doi.org/10.4143/> crt.2020.974.

[7] ZHOU H, XIN Y, LI S. A diabetes prediction model based on Boruta feature selection and ensemble learning [J]. BMC Bioinformatics, 2023, 24(1). <https://doi.org/10.1186/>s 12859-023-05300-5.

[8] SUN Y, ZHANG Q, YANG Q, et al. Screening of Gene Expression Markers for Corona Virus Disease 2019 Through Boruta_MCFS Feature Selection [J]. Frontiers in Public Health, 2022, 10. https://doi.org/10.3389/fpubh.2022.901602.

[9] MOORE A, BELL M. XGBoost, A Novel Explainable AI Technique, in the Prediction of Myocardial Infarction: A UK Biobank Cohort Study [J]. Clinical Medicine Insights: Cardiology, 2022, 16. <https://doi.org/10.1177/> 11795468221133611.

[10] XU J, TANG B, HE H, MAN H. Semisupervised Feature Selection Based on Relevance and Redundancy Criteria [J]. IEEE Transactions on Neural Networks and Learning Systems, 2017, 28(9): 1974-84. <https://doi.org/10.1109/> TNNLS.2016.2562670.
