# Supplementary material for: An Interpretable Machine Learning Model for Predicting the Presence of Talaromycosis in HIV Patients Lacking Skin Lesions
Source: Mycopathologia. 2026 Jul 21;191(4):66. doi: 10.1007/s11046-026-01089-y (PMC13384986; doi:10.1007/s11046-026-01089-y)
Supplement: Supplementary file 1 — Supplementary file1 (ZIP 1622 KB) [file 11046_2026_1089_MOESM1_ESM.zip › ESM/Supplementary Methods 2.docx]

**Feature Selection Using Multiple Machine Learning Algorithms**

In this study, the Random Forest (RF) algorithm was employed to generate an ensemble comprising 100 decision trees, utilizing random splitting at each node and a predetermined random seed (seed=42) to ensure reproducibility. The out-of-bag (OOB) error rate was calculated to be 22.8%, reflecting a relatively low error rate on the OOB samples and suggesting robust generalization capabilities. Notably, the model exhibited particularly low error rates in the classification of non-*Talaromyces marneffei* infection cases, thereby further substantiating its efficacy for this specific category. (Supplementary Fig. S3). Compared with the Mean Decrease Accuracy, the Mean Decrease Gini (MDG) is better suited for handling high-dimensional or noisy datasets [1]. Therefore, our study chose MDG. The 36 predictive variables influencing talaromycosis diagnosis in the training set were ranked in descending importance. (Supplementary Fig. S4).

In the Lasso regression model, 18 predictors retained non-zero coefficients when the minimal lambda (λ_min_) was specified as 0.016. The variable selection process was systematically evaluated through penalization path analysis (Fig. 2) and coefficient trajectory visualization (Fig. 3). Notably, the model bias exhibited negligible fluctuations across the regularization interval spanning from λ1se to λ_min_, indicating stable parameter estimation within this critical range of penalty values [2]. Supplementary Figure S5 displays the ranking of nonzero predictive variables. The coefficient analysis indicated that peripheral or abdominal lymphadenopathy (POAL) possessed the highest absolute coefficient value (β = 0.263, positive), signifying its primary role as the most significant determinant of the target variable. Subsequently, absolute lymphocyte count (ALC) displayed a considerable negative relationship with the outcome (β = -0.038). Conversely, variables such as aspartate aminotransferase (AST) exhibited coefficients nearing zero, suggesting a minimal impact on the model's predictive capability. The Boruta algorithm, a random forest-based feature selection technique, employs permutation importance analysis to iteratively eliminate insignificant predictors while maintaining both strong and moderate associations with clinical outcomes [3]. The algorithm identified 12 significant variables after 20 iterations. Supplementary Figs. S6 and S7 illustrate the fluctuations in variable importance scores during the Boruta process and the shifts in feature importance rankings across various classifier executions. The computational efficiency of Extreme Gradient Boosting (XGBoost) facilitates accurate model training with limited data, and its robust generalization and scalability render it particularly suitable for medical data analysis, thereby enhancing the reliability of diagnostic and prognostic outcomes [4]. Ultimately, A total of 32 predictor variables were identified, with their significance depicted in Supplementary Fig. S8. Mutual Information (MI) enables distribution-free detection of non-linear relationships, facilitating robust feature selection without requiring data distribution assumptions. It effectively identifies complex feature interactions and handles mixed data types [5], conclusively identifying 31 predictive features with high discriminative power from an initial set of 36 candidate variables. The feature importance is shown in Supplementary Fig. S9.

The shared features for model construction were the predictor variables identified through RF, XGBoost, Lasso, MI, and Boruta, which included 12 variables: albumin (ALB), ALC, Hemoglobin (HB), alanine aminotransferase (ALT), AST/ALT ratio, C-reactive protein (CRP), white blood cell count (WBC), platelet count (PLT), POAL, CD^4^+ T-cell count levels, AST, and age. For further details, please refer to Figure 4 for the Venn diagram and Supplementary Table S1.

**References**

[1] GREENER J G, KANDATHIL S M, MOFFAT L, JONES D T. A guide to machine learning for biologists [J]. Nature Reviews Molecular Cell Biology, 2021, 23(1): 40-55. https://doi.org/10.1038/ s41580-021-00407-0.

[2] YAQI W, YIWEI S, YIMIN L. Prognosis prediction of paraquat poisoning with lasso-logistic regression [J]. Occup Health & Emerg Rescue, 2022, 40(3): 259-64. https://doi.org/10.16369/j.oher.issn.1007-1326.2022.03.001.

[3] HAMIDI F, GILANI N, ARABI BELAGHI R, et al. Identifying potential circulating miRNA biomarkers for the diagnosis and prediction of ovarian cancer using the machine-learning approach: application of Boruta [J]. Frontiers in Digital Health, 2023, 5. https://doi.org/10.3389/fdgth.2023.1187578.

[4] GUAN X, DU Y, MA R, et al. Construction of the XGBoost model for early lung cancer prediction based on metabolic indices [J]. BMC Medical Informatics and Decision Making, 2023, 23(1). https://doi.org/10.1186/s12911-023-02171-x.

[5] WANG X, ZHOU Y, IQBAL N. Multi-Label Feature Selection with Conditional Mutual Information [J]. Computational Intelligence and Neuroscience, 2022, 2022: 1-13. https://doi.org/10.1155/2022/9243893.
