# Supplementary material for: An Interpretable Machine Learning Model for Predicting the Presence of Talaromycosis in HIV Patients Lacking Skin Lesions
Source: Mycopathologia. 2026 Jul 21;191(4):66. doi: 10.1007/s11046-026-01089-y (PMC13384986; doi:10.1007/s11046-026-01089-y)
Supplement: Supplementary file 1 — Supplementary file1 (ZIP 1622 KB) [file 11046_2026_1089_MOESM1_ESM.zip › ESM/Supplementary Table S1 The process of feature screening.docx]

**Table S 1 Identification of Key Variables by Five Feature Screening Algorithms and Their Intersection in Predictive Modeling**

| **Variable screening** | **key variables** |
| --- | --- |
| Random Forest selected features | AST、ALC、POAL、CRP、PLT、ALB、WBC、Hb、ALT、CD4、Age (years)、AST/ALT、BUN、Cr、BMI、G、Poor appetite、Hepatomegaly、Fever、Occupation、Pneumocystis pneumonia、Marital status、Oral candidiasis、Splenomegaly、Cough、Bacterial Pneumonia、Sex、Tuberculosis、Nationality、Hepatitis B、Cytomegalovirus、Herpesvirus、Injection drug user、Hepatitis C、cryptococcosis、 Lymphoma. |
| Lasso selected features | POAL、Poor appetite、AST/ALT、Fever、CRP、ALT、Cr、AST、G、PLT、CD4、BMI、Hb、Age (years)、WBC、BUN、ALB、 ALC. |
| Boruta selected features | Age (years)、POAL、Hb、PLT、WBC、ALC、CRP、AST、ALT、AST/ALT、ALB、CD4. |
| XGBoost selected features | AST、POAL、Hepatitis C、Cytomegalovirus、CRP、Poor appetite、ALC、Tuberculosis、Nationality、PLT、 WBC、Bacterial Pneumonia、CD4、Age (years)、ALB、BMI、Hepatitis B、G、Injection drug user、Hb、Pneumocystis pneumonia、BUN、AST/ALT、  Cr、Cough、ALT、Fever、Oral candidiasis、Marital status、Occupation、Hepatomegaly、Sex. |
| Mutual Information selected features | CRP、AST、ALC、G、PLT、ALB、POAL、CD4、Splenomegaly、ALT、BMI、cryptococcosis、Poor appetite、Hb、Bacterial Pneumonia、 Cr、Lymphoma、Hepatomegaly、BUN、Fever、AST/ALT、Hepatitis B、Cytomegalovirus、Nationality、Pneumocystis pneumonia、Occupation、Age (years)、Injection drug user、WBC、Sex、Marital status. |
| Intersection features | CD4、AST/ALT、ALT、Hb、CRP、ALB、POAL、ALC、Age (years)、WBC、PLT、AST. |

Abbreviations: BMI, Body mass index; POAL, Peripheral or abdominal lymphadenopathy; HB, Hemoglobin; WBC, White blood cell; ALC, Absolute lymphocyte count; CRP, C-reactive protein; PLT, Platelet; AST, Aspartate aminotransferase; ALT, Alanine transaminase; ALB, albumin; BUN, Blood urea nitrogen; G, (1-3)-β-D glucan; CD4, CD4^+^ T-cell count levels.
