# Supplementary material for: An Interpretable Machine Learning Model for Predicting the Presence of Talaromycosis in HIV Patients Lacking Skin Lesions
Source: Mycopathologia. 2026 Jul 21;191(4):66. doi: 10.1007/s11046-026-01089-y (PMC13384986; doi:10.1007/s11046-026-01089-y)
Supplement: Supplementary file 1 — Supplementary file1 (ZIP 1622 KB) [file 11046_2026_1089_MOESM1_ESM.zip › ESM/Supplementary Table S2 Diagnostic performance of models.docx]

**Table S2 Diagnostic performance of different models for predicting TM infection in training and test cohorts**

| Model | Cohort | AUC (95% CI) | ACC (95% CI) | Sensitivity | Specificity | PPV | NPV | MCC | F1_-_Score |
| --- | --- | --- | --- | --- | --- | --- | --- | --- | --- |
| NB | Training | 0.815(0.7863 - 0.8410) | 0.750(0.7198 - 0.7796) | 0.695 | 0.805 | 0.619 | 0.805 | 0.503 | 0.737 |
|  | Testing | 0.873(0.8219 - 0.9160) | 0.782(0.7386 - 0.7257) | 0.733 | 0.835 | 0.733 | 0.835 | 0.570 | 0.778 |
|  | External validation | 0.859(0.8162 - 0.9012) | 0.800(0.7656 - 0.8344) | 0.766 | 0.823 | 0.766 | 0.823 | 0.587 | 0.757 |
| MLP | Training | 0.534(0.4925 - 0.5709) | 0.491(0.4562 - 0.5252) | 0.619 | 0.360 | 0.619 | 0.360 | 0.122 | 0.551 |
|  | Testing | 0.730(0.7198 - 0.7796) | 0.663(0.7198 - 0.7796) | 0.619 | 0.711 | 0.619 | 0.711 | 0.331 | 0.764 |
|  | External validation | 0.656(0.5940 - 0.7134) | 0.541(0.4982 - 0.5838) | 0.565 | 0.525 | 0.565 | 0.525 | 0.088 | 0.50 |
| DT | Training | 1.000(1.0000 - 1.0000) | 0.999(0.9963 - 1.0000) | 0.998 | 1.000 | 0.998 | 1.000 | 0.998 | 0.999 |
|  | Testing | 0.770(0.7144 - 0.8238) | 0.772(0.7280 - 0.8165) | 0.819 | 0.722 | 0.819 | 0.722 | 0.544 | 0.789 |
|  | External validation | 0.699(0.6437 - 0.7478) | 0.689(0.6487 - 0.7283) | 0.750 | 0.646 | 0.750 | 0.646 | 0.390 | 0.662 |
| KNN | Training | 0.860(0.8360 - 0.8839) | 0.778（0.7495- 0.8069） | 0.7985 | 0.7575 | 0.799 | 0.758 | 0.557 | 0.7841 |
|  | Testing | 0.751(0.6857 - 0.8126) | 0.663(0.6135 - 0.7796) | 0.695 | 0.629 | 0.695 | 0.629 | 0.325 | 0.682 |
|  | External validation | 0.812(0.7625 - 0.8582) | 0.715(0.6760- 0.7535) | 0.7581 | 0.6851 | 0.758 | 0.685 | 0.435 | 0.684 |
| LR | Training | 0.830(0.8022 - 0.8603) | 0.761（0.7314- 0.7903） | 0.725 | 0.798 | 0.725 | 0.798 | 0.524 | 0.7535 |
|  | Testing | 0.894(0.8481 - 0.9347) | 0.817(0.7760 - 0.8577) | 0.791 | 0.845 | 0.791 | 0.845 | 0.636 | 0.818 |
|  | External validation | 0.867(0.8244 - 0.9069) | 0.807(0.7726 - 0.8405) | 0.823 | 0.796 | 0.823 | 0.796 | 0.610 | 0.776 |
| RF | Training | 1.000(1.0000 - 1.0000) | 0.999(0.9963 - 1.0000) | 0.998 | 1.000 | 0.998 | 1.000 | 0.998 | 0.999 |
|  | Testing | 0.891(0.8483 - 0.9313) | 0.782(0.7386 - 0.8257) | 0.733 | 0.835 | 0.733 | 0.835 | 0.570 | 0.778 |
|  | External validation | 0.869(0.8243 - 0.9105) | 0.810(0.7761 - 0.8435) | 0.847 | 0.785 | 0.847 | 0.785 | 0.621 | 0.784 |
| SVM | Training | 0.809(0.7780 - 0.8382) | 0.714(0.6813 - 0.7437) | 0.631 | 0.795 | 0.631 | 0.795 | 0.432 | 0.689 |
|  | Testing | 0.839(0.7807 - 0.8921) | 0.777(0.7333 - 0.8211) | 0.733 | 0.825 | 0.733 | 0.825 | 0.559 | 0.774 |
|  | External validation | 0.921(0.8889 - 0.9505) | 0.853(0.8220 - 0.8829) | 0.823 | 0.873 | 0.823 | 0.873 | 0.695 | 0.819 |
| XGB | Training | 1.000(1.0000 - 1.0000) | 0.999(0.9963 - 1.0000) | 1.000 | 0.998 | 1.000 | 0.998 | 0.998 | 0.999 |
|  | Testing | 0.878(0.8302 - 0.9213) | 0.812(0.7706 - 0.8531) | 0.819 | 0.804 | 0.819 | 0.804 | 0.623 | 0.819 |
|  | External validation | 0.826(0.7798 - 0.8706) | 0.7607(0.7240 - 0.7973) | 0.742 | 0.774 | 0.742 | 0.774 | 0.511 | 0.716 |

Abbreviations: NB, Naive Bayes; MLP, Multilayer Perceptron; DT, Decision Tree; KNN, K-Nearest Neighbors; LR, Logistic Regression; RF, Random Forest; SVM, Support Vector Machine; XGB, Extreme Gradient Boosting; ACC, Accuracy.
