# Supplementary figures and images for: An Interpretable Machine Learning Model for Predicting the Presence of Talaromycosis in HIV Patients Lacking Skin Lesions
Source: Mycopathologia. 2026 Jul 21;191(4):66. doi: 10.1007/s11046-026-01089-y (PMC13384986; doi:10.1007/s11046-026-01089-y)

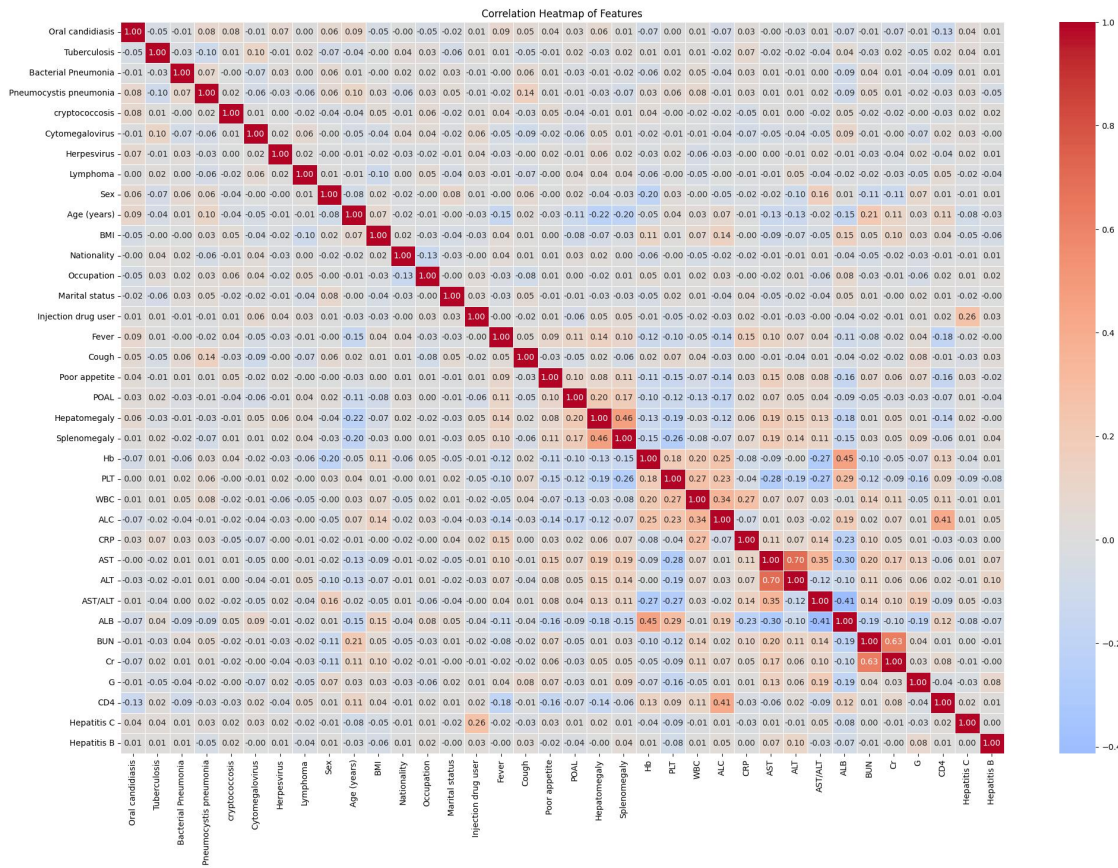

Supplement: Supplementary file 1 — Supplementary file1 (ZIP 1622 KB) [file 11046_2026_1089_MOESM1_ESM.zip › ESM/Supplementary Fig S1 Correlation Heatmap of Feature.pdf]

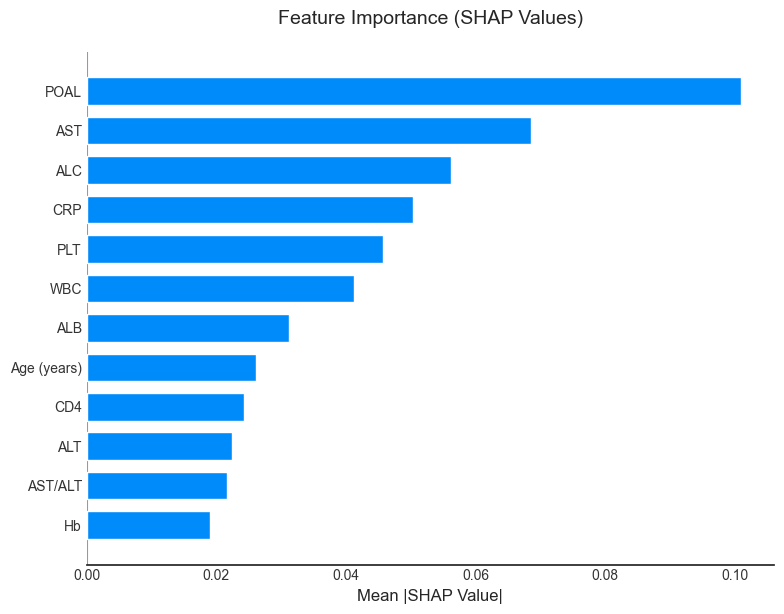

Supplement: Supplementary file 1 — Supplementary file1 (ZIP 1622 KB) [file 11046_2026_1089_MOESM1_ESM.zip › ESM/Supplementary Fig S10 Feature lmportance of SHAP Values.TIFF]

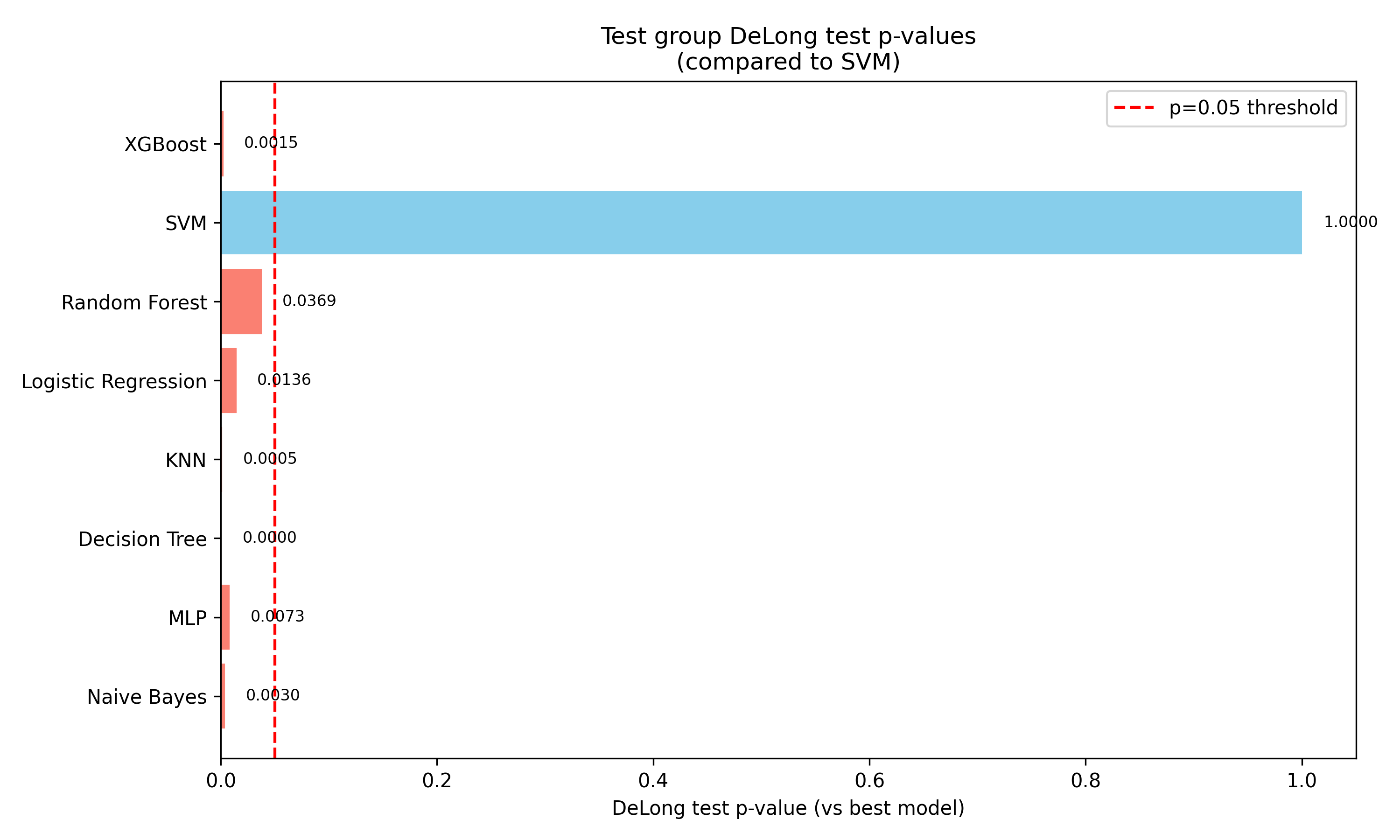

Supplement: Supplementary file 1 — Supplementary file1 (ZIP 1622 KB) [file 11046_2026_1089_MOESM1_ESM.zip › ESM/Supplementary Fig S12 Test_group_Delong.TIFF]

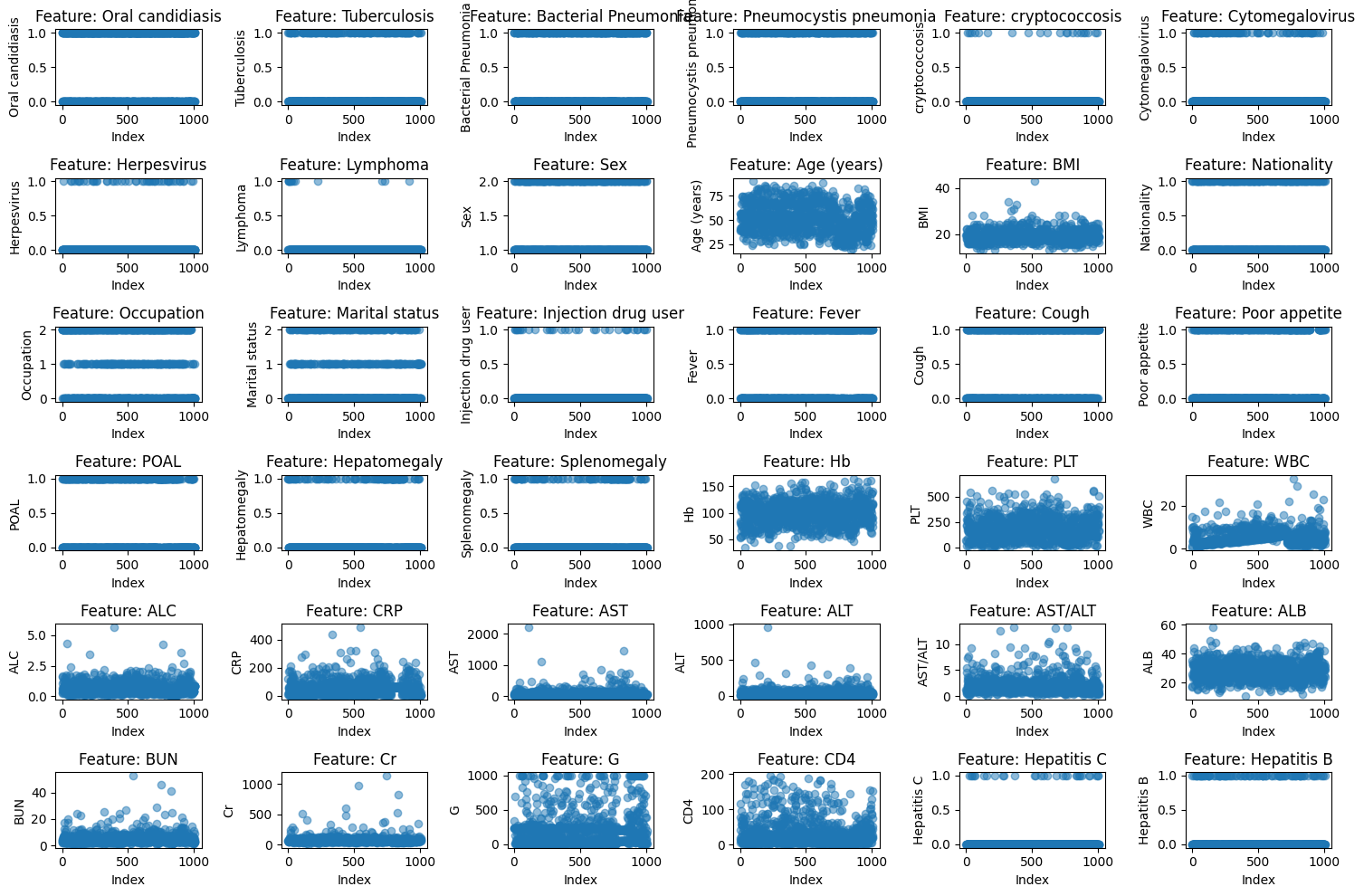

Supplement: Supplementary file 1 — Supplementary file1 (ZIP 1622 KB) [file 11046_2026_1089_MOESM1_ESM.zip › ESM/Supplementary FIG S2 the distribution of 36 Variables.TIFF]

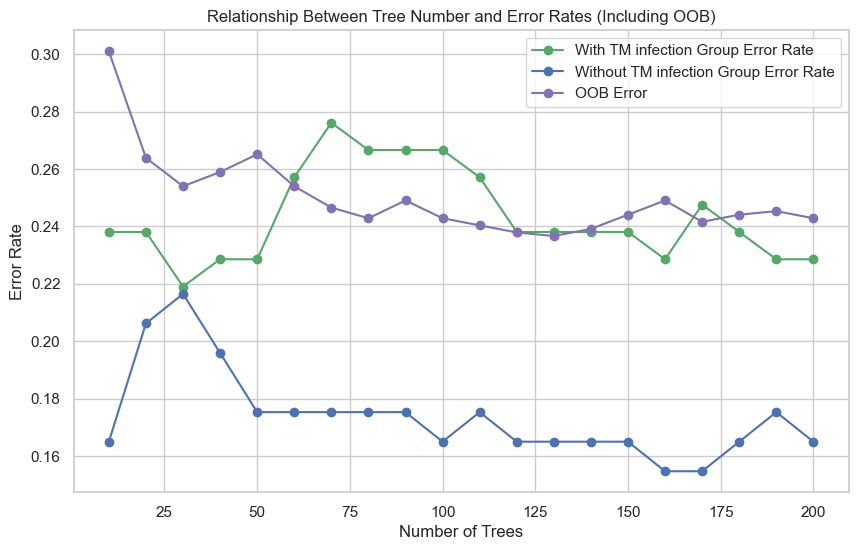

Supplement: Supplementary file 1 — Supplementary file1 (ZIP 1622 KB) [file 11046_2026_1089_MOESM1_ESM.zip › ESM/Supplementary Fig S3.TIFF]

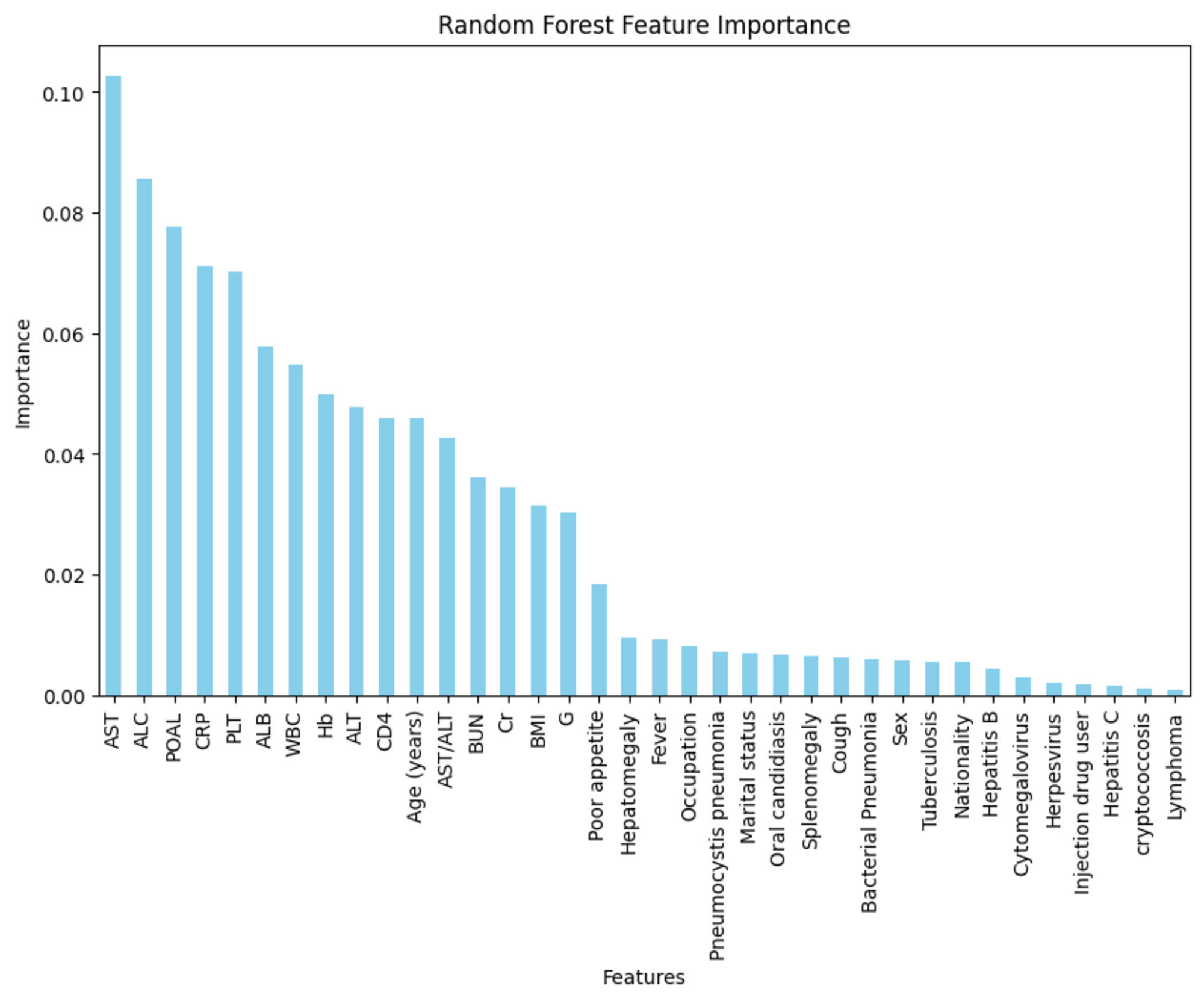

Supplement: Supplementary file 1 — Supplementary file1 (ZIP 1622 KB) [file 11046_2026_1089_MOESM1_ESM.zip › ESM/Supplementary Fig S4 List 36 predictive variables.tiff]

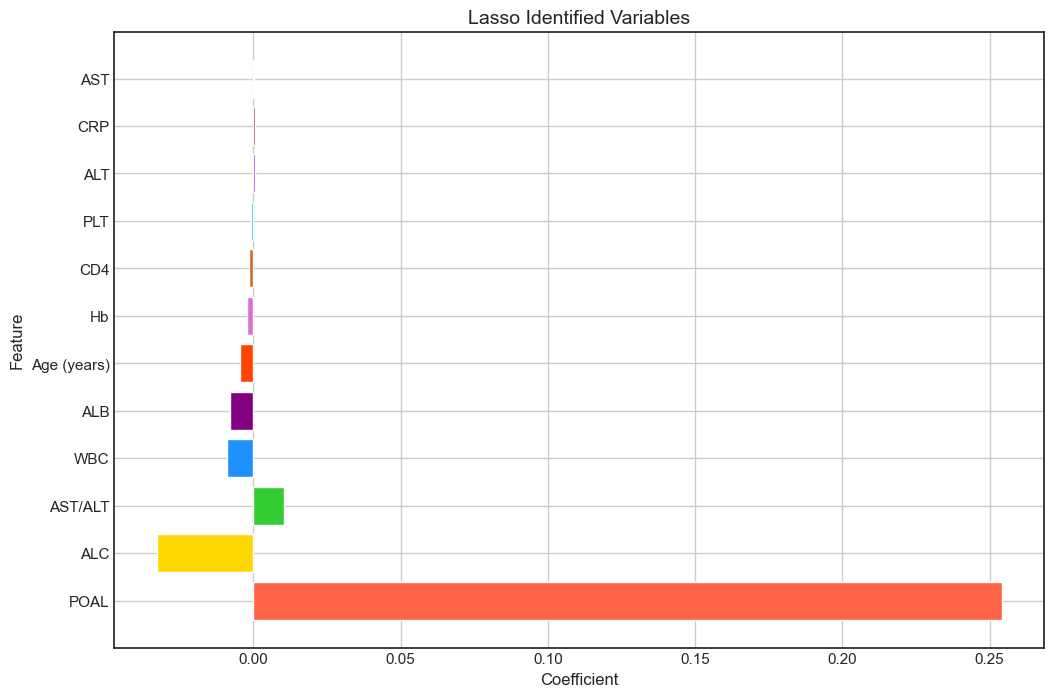

Supplement: Supplementary file 1 — Supplementary file1 (ZIP 1622 KB) [file 11046_2026_1089_MOESM1_ESM.zip › ESM/Supplementary Fig S5 12 non zero predictive variables.TIFF]

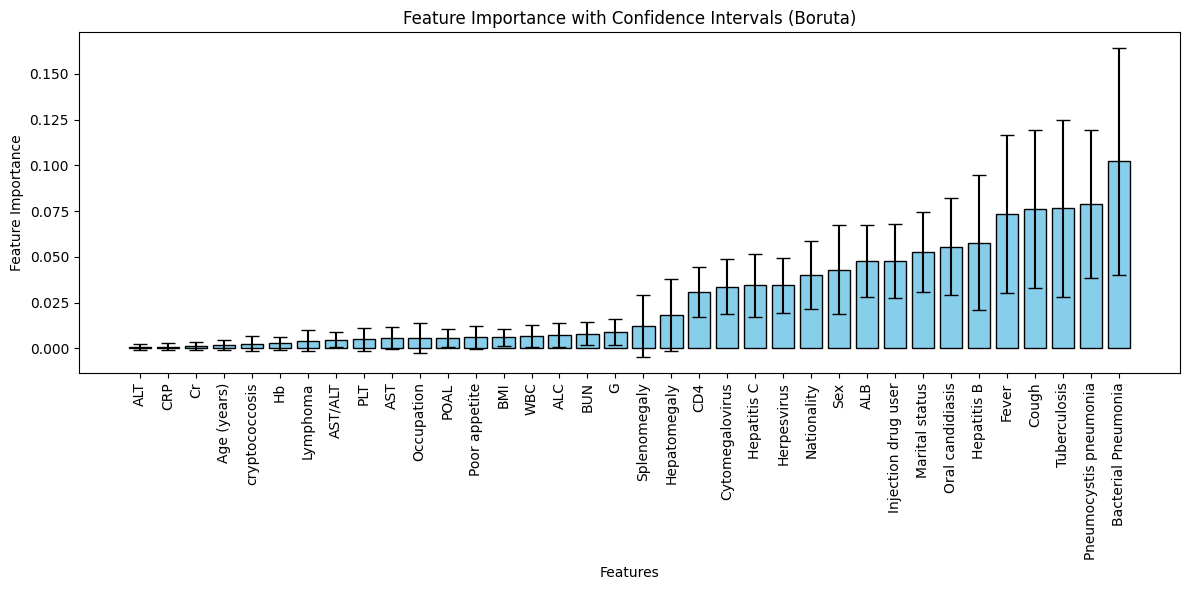

Supplement: Supplementary file 1 — Supplementary file1 (ZIP 1622 KB) [file 11046_2026_1089_MOESM1_ESM.zip › ESM/Supplementary Fig S6 Feature Importance.TIFF]

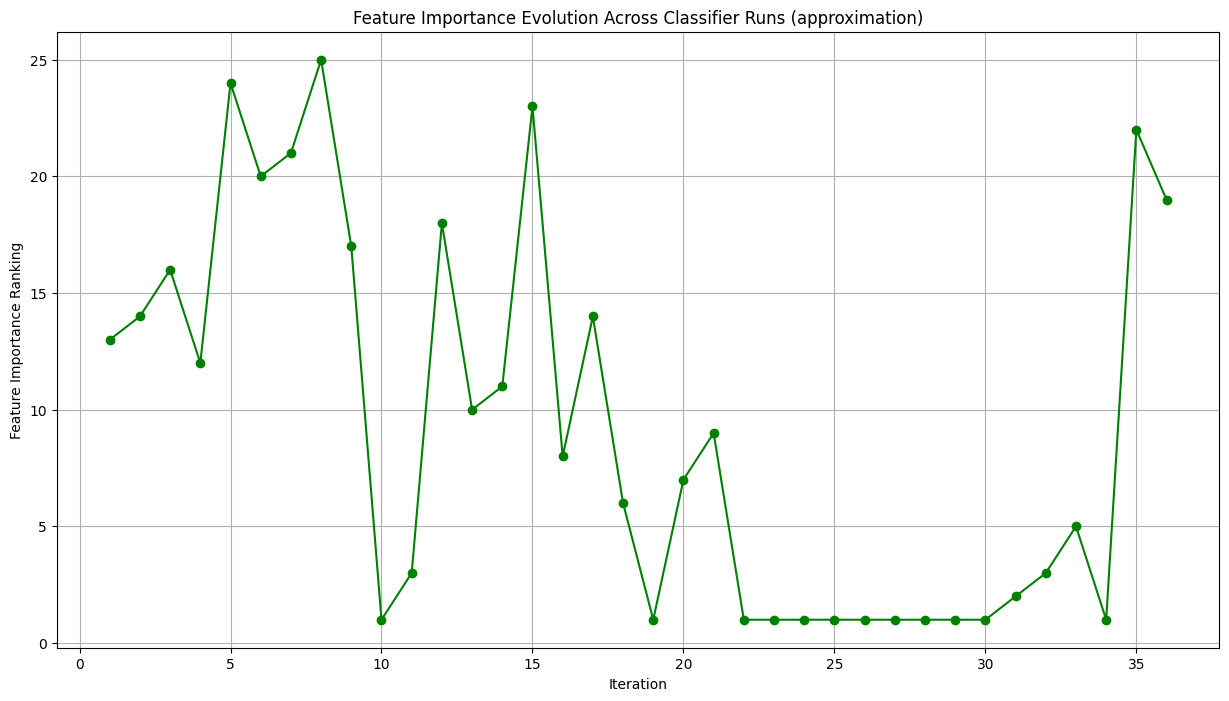

Supplement: Supplementary file 1 — Supplementary file1 (ZIP 1622 KB) [file 11046_2026_1089_MOESM1_ESM.zip › ESM/Supplementary Fig S7 Feature Importance Ranking.TIFF]

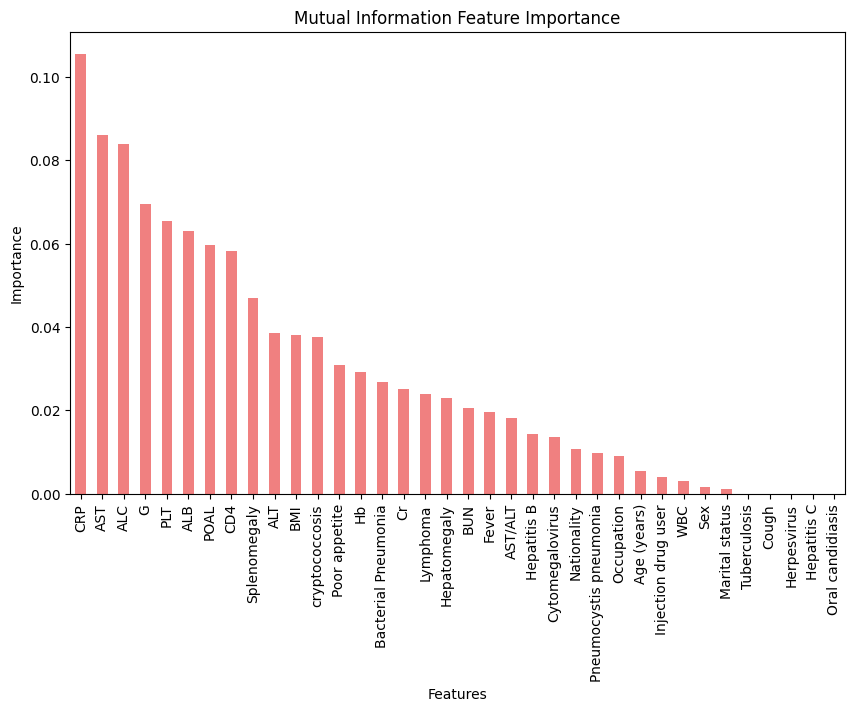

Supplement: Supplementary file 1 — Supplementary file1 (ZIP 1622 KB) [file 11046_2026_1089_MOESM1_ESM.zip › ESM/Supplementary Fig S8 The variables selected by MIt .TIFF]

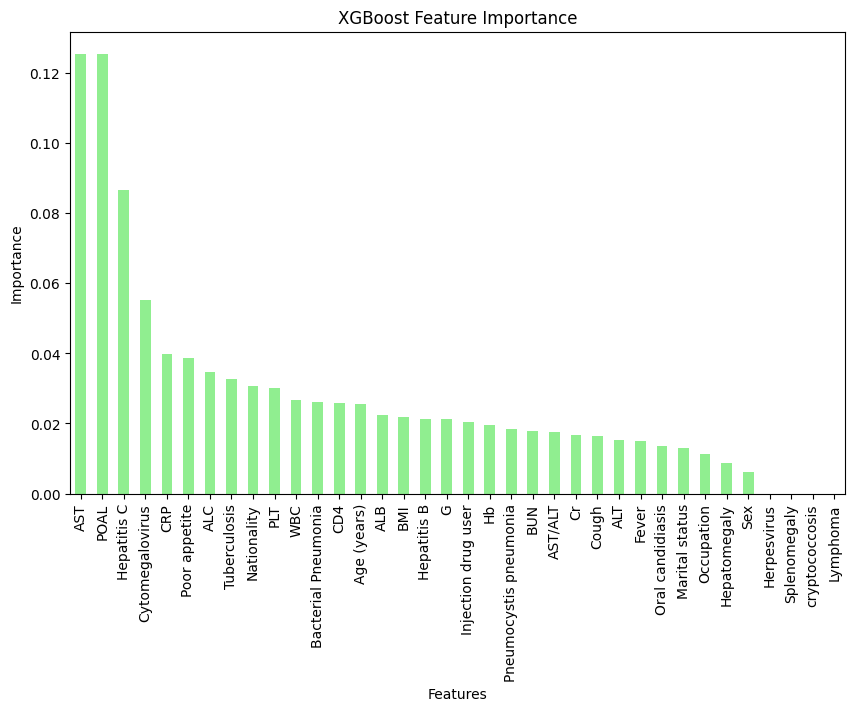

Supplement: Supplementary file 1 — Supplementary file1 (ZIP 1622 KB) [file 11046_2026_1089_MOESM1_ESM.zip › ESM/Supplementary Fig S9 The variables selected by XGBoost .TIFF]
